# Detection of respiratory frequency rhythm in human alpha phase shifts: topographic distributions in wake and drowsy states

**DOI:** 10.3389/fphys.2024.1511998

**Published:** 2025-01-06

**Authors:** Aleksandar Kalauzi, Zoran Matić, Edin Suljovrujić, Tijana Bojić

**Affiliations:** ^1^ Department for Life Sciences, Institute for Multidisciplinary Research, University of Belgrade, Belgrade, Serbia; ^2^ Laboratory for Radiation Chemistry and Physics-030, Institute for Nuclear Sciences Vinča-National Institute of the Republic of Serbia, University of Belgrade, Belgrade, Serbia

**Keywords:** respiration, electroencephalography, alpha activity, phase shift, wake and drowsy, mind–body interaction

## Abstract

**Introduction:**

The relationship between brain activity and respiration is recently attracting increasing attention, despite being studied for a long time. Respiratory modulation was evidenced in both single-cell activity and field potentials. Among EEG and intracranial measurements, the effect of respiration was prevailingly studied on amplitude/power in all frequency bands.

**Methods:**

Since phases of EEG oscillations received less attention, we applied our previously published carrier frequency (*CF*) mathematical model of human alpha oscillations on a group of 10 young healthy participants in wake and drowsy states, using a 14-channel average reference montage. Since our approach allows for a more precise calculation of *CF* phase shifts (*CFPS*) than any individual Fourier component, by using a 2-s moving Fourier window, we validated the new method and studied, for the first time, temporal waveforms *CFPS*(*t*) and their oscillatory content through FFT (*CFPS*(*t*)).

**Results:**

Although not appearing equally in all channel pairs and every subject, a clear peak in the respiratory frequency region, 0.21–0.26 Hz, was observed (max at 0.22 Hz). When five channel pairs with the most prominent group averaged amplitudes at 0.22 Hz were plotted in both states, topographic distributions changed significantly—from longitudinal, connecting frontal and posterior channels in the wake state to topographically split two separate regions—frontal and posterior in the drowsy state. In addition, in the drowsy state, 0.22-Hz amplitudes decreased for all pairs, while statistically significant reduction was obtained for 20/91 (22%) pairs.

**Discussion:**

These results potentially evidence, for the first time, the respiratory frequency modulation of alpha phase shifts, as well as the significant impact of wakeful consciousness on the observed oscillations.

## 1 Introduction

Breathing, one of the fundamental life rhythms, occurs at a frequency of approximately 0.25 Hz in humans at rest and consists of active inspiration and mostly passive expiration (Fleming et al., 2011). For many years, the relationship between brain activity and respiration has attracted researchers’ attention, and different quantitative measures, referring to this relationship, have been applied (Harper et al., 1998; Heck et al., 2017; Herrero et al., 2018; Del Negro et al., 2018; McKay et al., 2003; Nakamura et al., 2023). Due to its nature, respiration could be easily tracked and recorded, while the brain acts as an incomparably richer source of information, resulting in numerous methods applied to monitor its respiration-related activity. In addition to electroencephalography (EEG), most common methods include (BOLD) fMRI (Damoiseaux et al., 2006; Jung and Kang, 2021), intracortical recording (Viczko et al., 2014; Väyrynen et al., 2023), or magnetoencephalography (MEG) (Hsu et al., 2020; Kluger and Gross, 2020). Within that framework, EEG oscillations are the most relevant approach, at least for our research. Although several studies have been conducted to investigate the relationship between EEG oscillations and respiratory signals, it is still unclear how respiration and human alpha activity are related. In a recent review paper, Brændholt et al. (2023) presented a list that included authors, objects (models), frequency, and brain regions. Among all the frequency bands analyzed (LF, delta, theta, alpha, beta1, beta2, and gamma), only three studies refer to human alpha activity: (Perl et al., 2019) EEG alpha and beta, the parietal cortex, and hippocampus; (Kluger and Gross, 2021) MEG delta, theta, alpha, beta, gamma; cortical/subcortical structures, and (Kluger et al., 2021) MEG alpha and parieto-occipital cortex. However, even in studies where the alpha activity was registered, the measured quantity was mostly amplitude or power (Bing-Canar et al., 2016; Herrero et al., 2018; Perl et al., 2019; Kluger et al., 2021). Since their discovery, alpha waves are maybe the most studied cortical oscillations. Current research suggests that they are generated by inhibitory thalamic interconnection, thalamo-cortical feedback loops, and cortico-cortical networks (Da Silva et al., 1980; Nunez et al., 2001). According to new research, cortical structures lead the thalamus, while alpha propagates from higher-order to lower-order areas in both the visual and somatosensory cortex (Halgren et al., 2019). Its amplitude is inversely proportional to the level of cortical activity; strong alpha activity is associated with cortical and behavioral deactivation (e.g., Klimesch, 1999; Klimesch et al., 2007; Rihs et al., 2007). Its involvement in perceptual and memory processes was also studied (Klimesch, 1999; Ergenoglu et al., 2004; Klimesch et al., 2005).

Phases of cortical oscillations play a significant role in brain information processing since they are related to the exact timing of neural activity (Sauseng and Klimesch, 2008). EEG phase synchronization shows communication between distant but functionally related neural populations, where information is exchanged between global and local neuronal networks, as well as the sequential temporal activity of neural processes in response to incoming sensory stimuli (Sauseng and Klimesch, 2008). The relationship between respiratory and EEG alpha phases was studied by Hsu et al. (2020). In their MEG study, the authors explored the effect of slow and normal-paced breathing (0.125 and 0.25 Hz, respectively) on the cosine similarity version of the inter-trial phase coherence (Tallon-Baudry et al., 1996) between respiratory and alpha phases. Based on simultaneous magnetoencephalography and respiratory measurements, they reported that while participants performed slow-paced compared to normal-paced breathing, this condition modulated alpha phase activity, allowing it to appear more organized across a wide range of brain areas. This suggested that slow-paced inspiration was able to organize the cortical alpha phase in a more regularized pattern than the phase associated with normal-paced breathing. Since we found no studies where a respiratory frequency rhythm directly modulates alpha phases (phase differences) in a strictly spontaneous breathing regime, we tried to attempt this research. In our previous papers (Kalauzi et al., 1998; Kalauzi et al., 2009; Kalauzi et al., 2012b; Kalauzi et al., 2018), we proposed, by applying the variable amplitude carrier frequency (*CF*) mathematical model for human alpha (or rat theta) activity, a procedure to calculate more precisely phase shifts (*PS*) between any two alpha activity EEG channels than using any Fourier component (FC) phase shift. In this work, for the first time, we applied fast Fourier transform (FFT) on *CFPS* time courses, FFT (*CFPS*(*t*)), in order to examine their oscillatory content.

Body–brain interactions in different states of alertness are recently gaining attention. In addition to the normal resting wake state, these interactions were studied in normal (Abdalbari et al., 2022) and disordered (Faes et al., 2016) sleep. In contrast, a network approach was applied to quantify the cardio–respiratory–EEG relationship in states of heightened alertness: information dynamics (storage and transfer; Zanetti et al., 2019) and multivariate correlation (Pernice et al., 2021). We could not find the analysis of these interactions in the state of drowsiness, a transient state between wakefulness and sleep. Moreover, although several papers address the dependence of human cognition on the respiratory phase (Perl et al., 2019), the topographic dependence of alpha inter-channel phase shift oscillations in different states of alertness was not studied. Therefore, after a detailed validation of the method, topographic distributions of the most prominent amplitudes from this analysis, calculated in wake and drowsy states, are presented and their difference is statistically compared. The state of drowsiness (or hypnagogic state) is divided into nine stages (Hori et al., 1990). Psychophysiological studies have revealed that the main characteristics of this state include decay of alertness, decreased attention, vigilance, and sensory signal detection (Naatanen and Picton, 1987; Ogilvie and Wilkinson, 1984; Ogilvie et al., 1991, respectively). Ogilvie and Wilkinson, (1984) discovered that the transition from wakefulness to sleep is a gradual process. Ogilvie et al. (1991) and Harsh et al. (1994) concluded that the first significant changes in the attention level occurred during Hori’s stages 2 and 3. These data suggest that the first half of the hypnagogic period (Hori’s stages 1–4, where stage 1 has maximal and stage 4 minimal alpha power, without other frequency bands), with its distinct characteristics of decreased attention and dream-like mentation (Nielsen et al., 2005), warrants particular scientific investigation. In our experiments, the state of alertness was monitored by a neurologist who prevented subjects from transitioning to S1 of NREM sleep (N1), i.e., passing beyond Hori’s stage 4. This was confirmed by the fact that the two sets of spectra showed only alpha band amplitude attenuation, without any frequency shift (Bojić et al., 2010).

It should be noted that in this work, we hypothesize that the main rhythm depicted in the respiratory frequency range is caused by respiration. However, since we did not analyze concurrent EEG and respiratory recordings from the same group of individuals, at this stage, we cannot definitely confirm the hypothesis. Therefore, all considerations about body–brain interactions included in this work should be considered conditional, assuming that our hypothesis is correct.

## 2 Methods

### 2.1 Subjects, experimental protocol, and data preparation

#### 2.1.1 EEG recordings

Signals analyzed in this work were originally recorded by Vuckovic et al. (2002) and used in our previous works (Bojić et al., 2010; Kalauzi et al., 2012a; 2012b; 2015; 2018). All recordings were performed in accordance with the medical ethical standards after the subjects signed the informed consent form approved by the local ethical committee. Ten adult healthy human subjects (seven male and three female individuals), age 25–35 (mean ± SD = 28.3 ± 6.5) years, of normal intelligence and without mental disorders were reported after passing a neurological screening. The subjects were lying in a dark room with their eyes closed (standard eyes closed no-task condition; Stam et al., 2002; Stam and van Dijk, 2002). A neurologist monitored their state of alertness and prevented them from falling asleep beyond S1 of NREM sleep. The participants were not previously subjected to sleep deprivation or deviation from their circadian cycles. They were not taking any medication. Based on a visual inspection, artifacts were removed manually. Other details about data collection and preparation can be found in Vuckovic et al. (2002). Two neurologists were independently classifying all signals into wake and drowsy periods. The study included only those sequences that were classified as clearly awake or drowsy by both experts (60 s for each state and each subject).

An EEG machine (MEDELEC 1A97 EEG system, MEDILOG BV, Nieuwkoop, the Netherlands) was used in an electromagnetically shielded room during each 30 min session. EEG electrodes (Ag/AgCl; impedances *R* _ 5 k_) were positioned at 14 locations (F7, F8, T3, T4, T5, T6, F3, F4, C3, C4, P3, P4, O1, and O2), following the International 10–20 system, with an average reference. The recordings were band-pass filtered between 0.5 and 70 Hz. The EEG recordings were digitized using 12-bit resolution and a sampling rate of 256 Hz per channel (A/D PCI board, Data Translation 2801, Marlboro, MA, USA).

#### 2.1.2 Respiratory signal recordings

We conducted the experimental protocol on 20 healthy adult human subjects (13 male and 7 female individuals), with mean ± SD = 34.4 ± 7.4. The protocol was approved by the Ethical Committee of the Faculty of Medicine, University of Belgrade (No. 2650/IV-24). The inclusion criteria are as follows: age between 20 and 45 years and absence of any health problems. The exclusion criteria are as follows: patients receiving any medical therapy; a history of pulmonary, cardiovascular, or any other disease at the time of or leading up to the experiments (such as cold, flu, pollen allergy, high temperature, and migraines); and the presence of pathological symptoms during the measurements (high blood pressure, arrhythmias, headache, fatigue, etc.). An additional criterion of exclusion for female participants was the second part of the menstrual cycle (because of its impact on cardiovascular autonomic regulation; Bai et al., 2009; Javorka et al., 2018). All participants were advised to refrain from food and drink for 4 h prior to the start of measurements, to relax and stay attentive, and not to exercise (running, gym, yoga, etc.). Five participants (out of 25) were excluded because of pathological symptoms being discovered during the recordings.

The protocol was executed under controlled laboratory conditions at the Laboratory for Biosignals, Institute for Biophysics, Faculty of Medicine, University of Belgrade. It was conducted between 8 and 12 a.m. in a quiet, refreshing environment at a constant temperature (22°C ± 1°C). All participants were subjected to 10 min of relaxation in a supine position before recording, with no restriction imposed on the air flow. Subjects were advised to adjust the ventilation at the most comfortable rate. They were also instructed not to talk during the recording. Respiration signals were recorded for 20 min. Acquisition was done using the BIOPAC MP100 system (BIOPAC System, Inc., Santa Barbara, CA, USA; AcqKnowledge 3.91 software). The belt with a resistive strain gauge transducer for the continuous recording of breathing was placed slightly above the costal line. All signals were sampled at a frequency rate of 1,000 Hz. We adjusted filters according to Biopac’s instructions for general measurements: gain setting 10, low pass filter with 10 Hz, and no high pass filter (DC-absolute respiratory measurement). Breath-to-breath (BB) intervals were calculated by subtracting the time coordinates of successive inspiration onsets.

### 2.2 Data analysis

All programs implementing methods described in this work are original and were developed in MATLAB 2010a (MathWorks Inc., Natick, MA, United States). Statistical tests (Wilcoxon matched-pairs test) were performed using STATISTICA 8.0 (Stat Soft Inc., Tulsa, OK, United States).

#### 2.2.1 A short reminder on the *CFPS* calculation

It is assumed that two oscillations, with equal stable frequencies but variable amplitudes, are being analyzed. The task of determining their phase shift, regardless of whether it be at a single point in time or during the whole recorded time course, is hampered by the fact that each of the two oscillations is spread across a frequency region. The question as to which FC phase shift to rely on is raised. As described in detail in our previous works (Kalauzi et al., 1998; Kalauzi et al., 2009; Kalauzi et al., 2012b; Kalauzi et al., 2018), there are two reasons why a single oscillatory activity, with a stable frequency but variable amplitude, occupies a range of frequencies (rather than only one) when subjected to a concrete FFT analysis. The first reason is that its frequency is positioned between two adjacent FCs’ frequencies (which is almost always the case) and which we conveniently named “inter-componentality.” The second reason originates from the variability of amplitudes, where this variability raises to two sidelobes in the amplitude spectrum (in radio communications termed double sideband amplitude modulation, DSBAM).

For the first cause, let us observe a cosine wave with constant frequency *f*_*c*_ = *ω*_*c*_/(2*π*) (usually termed carrier frequency, *CF*), constant amplitude *A*_*c*_, and initial phase *φ*_*c*_:

$$u\left(t\right)=A_{c}\cos\left(\omega_{c}t+\varphi_{c}\right).$$

If such a signal of duration *T* is subjected to FFT analysis, then *CF* can be expressed as

$$\omega_{c}=k\omega_{0}+\Delta \omega ,$$

where *ω*_*0*_ = 2π/*T* and *kω*_*0*_ denotes the nearest lower Fourier component frequency. The amplitude spectrum of the signal *u*(*t*) can then be expressed as

$$A_{m}\left(n\omega_{0}\right)=\sqrt{x_{n}^{2}+y_{n}^{2}},$$ (1)

where real and imaginary parts of the spectrum are

$$x_{n}=\frac{4A}{T}\frac{\left(k\omega_{0}+\Delta \omega \right)}{{\left(k\omega_{0}+\Delta \omega \right)}^{2}+{\left(n\omega_{0}\right)}^{2}}\cos\left(\varphi_{c}+\pi \frac{\Delta \omega }{\omega_{0}}\right)\sin\left(\pi \frac{\Delta \omega }{\omega_{0}}\right),$$ (2)

$$y_{n}=-\frac{4A}{T}\frac{\left({n\omega }_{0}\right)}{{\left(k\omega_{0}+\Delta \omega \right)}^{2}+{\left(n\omega_{0}\right)}^{2}}\sin\left(\varphi_{c}+\pi \frac{\Delta \omega }{\omega_{0}}\right)\sin\left(\pi \frac{\Delta \omega }{\omega_{0}}\right).$$ (3)

It is clear from Equations 1–3 that this single-frequency oscillation occupies the whole available frequency range, with the largest amplitudes occurring next to *ω*_*c*_ and decreasing as one moves away from it.

Subsequently, let us observe two such oscillations with equal carrier frequencies:

$$u_{c1}\left(t\right)=A_{c1}\cos\left(\omega_{c}t+\varphi_{c1}\right);\;u_{c2}\left(t\right)=A_{c2}\cos\left(\omega_{c}t+\varphi_{c2}\right).$$

Equations 1–3 yield the following phase shift spectrum:

$$\Delta \varphi \left(n\omega_{0}\right)=\tan^{-1}\left(\frac{n\omega_{0}S_{1}\omega_{c}C_{2}-\omega_{c}C_{1}n\omega_{0}S_{2}}{\omega_{c}C_{1}\omega_{c}C_{2}+n\omega_{0}S_{1}n\omega_{0}S_{2}}\right),$$

where

$$S_{i}=\sin\left(\varphi_{ci}+\pi \frac{\Delta \omega }{\omega_{0}}\right);\;C_{i}=\cos\left(\varphi_{ci}+\pi \frac{\Delta \omega }{\omega_{0}}\right);i=1,2.$$ (4)

Based on Equation 4, the expansion of *Δφ*(*nω*_*0*_) into Taylor series around *ω*_*c*_ shows that there is an approximately linear dependence of *Δφ*(*nω*_*0*_) on *nω*_*0*_ in the vicinity of *ω*_*c*_, with FC phase shift values being around the carrier frequency phase shift *Δφ*_*c*_ = *φ*_*c2*_ – *φ*_*c1*_ (Kalauzi et al., 2012b, Appendix B). “Being around” means that *Δφ*(*nω*_*0*_) – *Δφ*_*c*_ changes sign when *nω*_*0*_ – *ω*_*c*_ does. Therefore, by angularly averaging FC phase shifts around *ω*_*c*_, we can obtain a more accurate estimate of *CFPS*, or *Δφ*_*c*_, than by relying on any of the single FC phase shifts.

Regarding the second cause, *i.e.*, the influence of amplitude variability on FC phase shifts, let us again observe two *CF* signals, this time both with variable amplitudes, where amplitude variability is achieved by applying amplitude modulation on both signals:

$$u_{ci}\left(t\right)=\left[1+u_{mi}\left(t\right)\right]U_{ci}\cos\left(\omega_{c}t+\varphi_{ci}\right),i=1,2,$$ (5)

where *u*_*mi*_ stands for the two modulating functions. After expanding both modulating functions into Fourier series and keeping the first *n* FCs, Equation 5 can be approximated with

$$u_{ci}\left(t\right)\approx \left[1+\sum_{j=1}^{n}U_{mj,i}\cos\left(\omega_{mj}t+\varphi_{mj,i}\right)\right]U_{ci}\cos\left(\omega_{c}t+\varphi_{ci}\right),i=1,2,$$

where *U*_*mj,i*_, *ω*_*mj*_, and *φ*_*mj,i*_ stand for the amplitude, frequency, and initial phase of the *j*th FC of the *i*th modulating function, respectively (please note that the modulating FC frequency *ω*_*mj*_ does not depend on *i*).

After multiplication, if we transform cosine products into sums, we obtain

$$u_{ci}\left(t\right)\approx U_{ci}\cos\left(\omega_{c}t+\varphi_{ci}\right)+\frac{1}{2}\sum_{j=1}^{n}U_{mj,i}U_{ci}\left[\cos\left(\left(\omega_{c}+\omega_{mj}\right)t+\varphi_{ci}+\varphi_{mj,i}\right)+\cos\left(\left(\omega_{c}-\omega_{mj}\right)t+\varphi_{ci}-\varphi_{mj,i}\right)\right].$$ (6)

Notably, Equation 6 contains three terms: one component at *CF*, *ω*_*c*_, and two sidebands at *ω*_*c*_ + *ω*_*mj*_ and *ω*_*c*_–*ω*_*mj*_. Components at the two sidebands have initial phases *φ*_*ci*_ + *φ*_*mj,i*_ and *φ*_*ci*_–*φ*_*mj,i*_, with an opposite sign of the second term. Therefore, the *CF* initial phase, *φ*_*ci*_, can again be obtained by angularly averaging phases of FCs around the carrier frequency, *ω*_*c*_. The same is valid for two signals, where by angular averaging of FC phase shifts around *CF*, we can calculate *CFPS* more accurately than by relying on any of FC phase shifts. Finally, according to both Equations 4, 6, in order to calculate *CFPS* of two signals as accurately as possible, both causes of the signals occupying a frequency range can be overcome by the same procedure–angular averaging of FC phase shifts around the *CF*. Accuracy of this calculation is further increased if we perform a weighted averaging with FC amplitudes as weights.

#### 2.2.2 Angle range extension method

In our previous works, we treated *CFPS* as a random variable, working mostly with distributions and histograms and deriving basic statistical properties from them (Kalauzi et al., 2009; Kalauzi et al., 2012b). Exploring time dependence of this quantity, *CFPS*(*t*), and its FFT spectral properties was not studied until now. In order to do a successful FFT analysis, we must first introduce some new procedures for preprocessing *CFPS*(*t*). One important step in that direction is described in the following section.

In this work, we performed two FFT procedures: the first procedure (“primary”) on initial EEG recordings. Since the duration of each recording was 60 s, with a primary sampling frequency of 256/s, 15360 samples were generated. They were subjected to a moving window FFT analysis, using 2-s long overlapping moving windows that advanced at a 0.25 s step. With these parameters, for each subject and each EEG channel, 233 spectra could be obtained, producing *CFPS*(*t*) waveforms with the same number of points. Secondary FFT analysis was done on these waveforms, but because they were relatively short, each of the 910 *CFPS*(*t*) signals was analyzed as a whole, without any moving windows, but after previous detrending. Although we expected relatively slow *CFPS*(*t*) oscillations to appear, we chose overlapping windows in order to generate a sufficiently high secondary sampling frequency (4 Hz), allowing us to detect *CFPS*(*t*) oscillations of up to 2 Hz. Analysis parameters of both levels (primary and secondary) are summarized in Table 1.

**TABLE 1:** Summary of parameter values for the two levels of analysis.

	Primary	Secondary
Input signal	EEG	*CFPS*(*t*)
Output signal	*CFPS*(*t*)	FFT (*CFPS*(*t*))
Sampling frequency	256/s	4/s
Window length (FFT epoch)	2 s	(60–2)/0.25 + 1 = 233 spectra 233*0.25 = 58.25 s
Window step	0.25 s	No moving window, whole signal FFT

**FIGURE 1:**
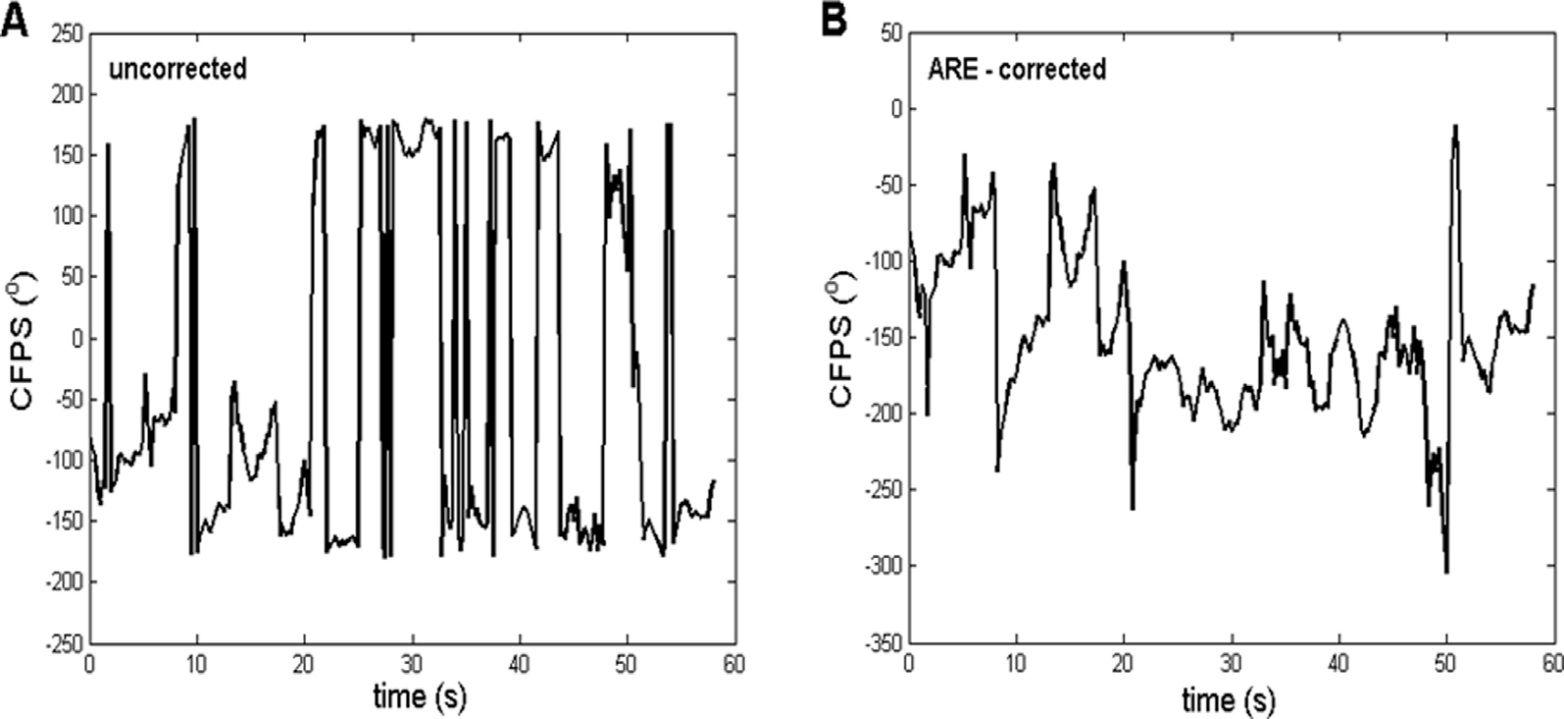
Representative example of the ARE method, in which the procedure, applied on the *CFPS*(*t*) waveform, had a substantial effect. **(A)** Uncorrected, derived from subject one between channels T3 and O2, where a contra-phase was present (*CFPS* values grouped approximately ±180°). **(B)** Corrected after applying the ARE procedure, where most of the “abrupt jumps” were eliminated, making it more suitable for further FFT analysis. The contra-phase is present usually when fronto-occipital or temporo-occipital pairs are being analyzed.

**FIGURE 2:**
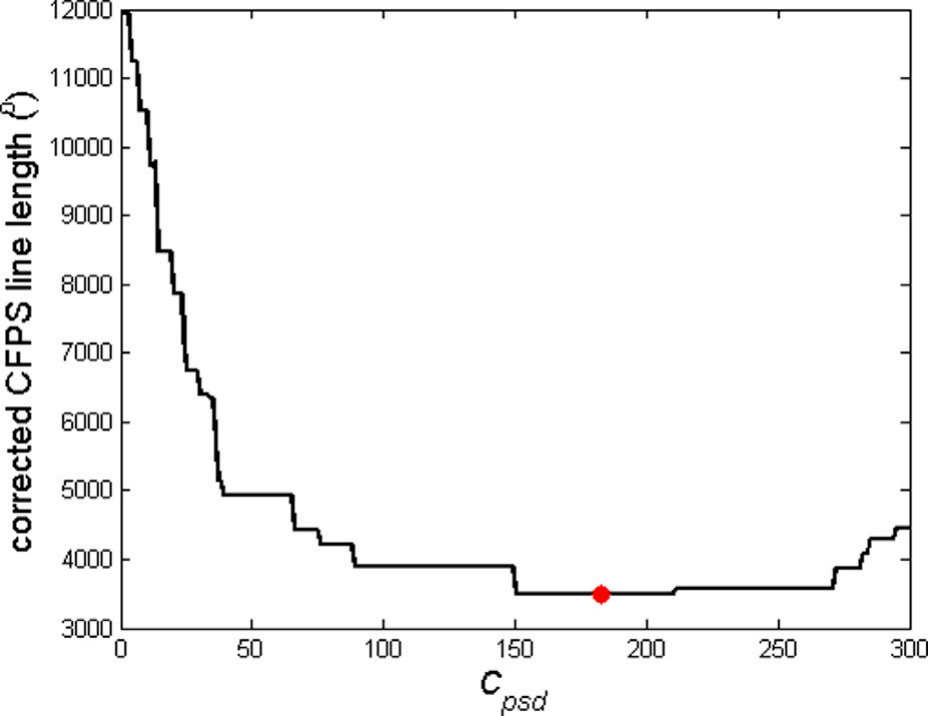
Example of dependence of the corrected *CFPS* line length on the critical phase shift difference, *C*_*psd*_. Optimal *C*_*psd*_, above which the ARE procedure is to be applied, corresponds to the minimal *CFPS* line length and is marked with a red dot. This example refers to the *CFPS* waveform presented in Figure 1A to produce the corrected waveform shown in Figure 1B.

When calculating formulas involving angles, it is often the case that the results are limited to the range of [-180°, 180°] or, alternatively, [0°, 360°]. An example of this fact is the often applied MATLAB atan2 command. In some physiological experiments dealing with oscillations, relatively small angle deviations around the zero baseline appear, requiring no correction of angle range. However, in many other cases, deviations of phases or phase-shifts of EEG signals are substantial. If time courses of angles are being recorded, a considerably artificial limitation to the abovementioned ranges causes frequent and big, abrupt jumps of angle values, as shown in angle time plots (Figure 1A). In order to achieve a more natural time dependence of angles, a correction procedure, named angle range extension (ARE), is proposed for the first time. This procedure is essentially aimed to detect these “abrupt jumps”, *i.e.*, differences between two adjacent *CFPS*(*t*) points, which are above a certain critical value (we named it “critical phase shift difference”, *C*_*psd*_). If *CFPS*(*i*) is the *CF* phase shift calculated at the *i*th Fourier window position, then if *CFPS*(*i*+1) < *CFPS*(*i*) and abs (*CFPS*(*i*+1) - *CFPS*(*i*)) > *C*_*psd*_, then *CFPSc*(*i*+1) *= CFPS*(*i*+1) +360°, where *CFPSc*(*i*+1) stands for the corrected value. Alternatively, if *CFPS*(*i*+1) > *CFPS*(*i*) and abs (*CFPS*(*i*+1) - *CFPS*(*i*)) > *C*_*psd*_, then *CFPSc*(*i*+1) *= CFPS*(*i*+1) −360°. Therefore, addition is performed in case of a sudden decrease and subtraction in case of an increase. However, determining the optimal value of *C*_*psd*_ remains a challenge. It can be assumed that the corrected *CFPS*(*i*) waveform should have a smaller line length, $\sum_{i}\text{abs}\left(CFPS\left(i+1\right)-CFPS\left(i\right)\right)$ , than the uncorrected line since long jumps, supposedly, were eliminated during the procedure. Therefore, for each waveform, we applied a series of ARE corrections, where *C*_*psd*_ was varied. For each *C*_*psd*_, the corrected line length was calculated, and *C*_*psd*_, where the line had a minimal length, was taken as the optimal value (Figure 2).

#### 2.2.3 FFT analysis of carrier frequency phase shifts, *CFPS*(*t*)

In our previous work (Kalauzi et al., 2012b), we studied statistical properties of *CFPS* of 10 test individuals in the wake and drowsy states, treating it as a random variable. In this study, we aimed at investigating probability distributions of phase shifts or phase potentials, from which properties such as the average value, most probable value, and standard deviation could be derived. At that stage, we did not analyze oscillatory properties of *CFPS*(*t*) waveforms, which could yield information about possible links of EEG with other physiological rhythms, such as ECG and respiration (so-called “mind–body” relationship).

Currently, we tried to combine two methods:

a) By treating alpha EEG activity as a carrier-stable inter-componential frequency and amplitude variable signal, we perform *CFPS* calculation, integrating Fourier component phase shifts from the whole alpha frequency range (8–12 Hz), according to the procedure described in our previous work (Kalauzi et al., 2012b).
b) On these “raw” *CFPS* waveforms, we apply the ARE method in order to obtain the waveforms suitable for further FFT analysis (parameters shown in Table 1).

#### 2.2.4 Method validation

**FIGURE 3:**
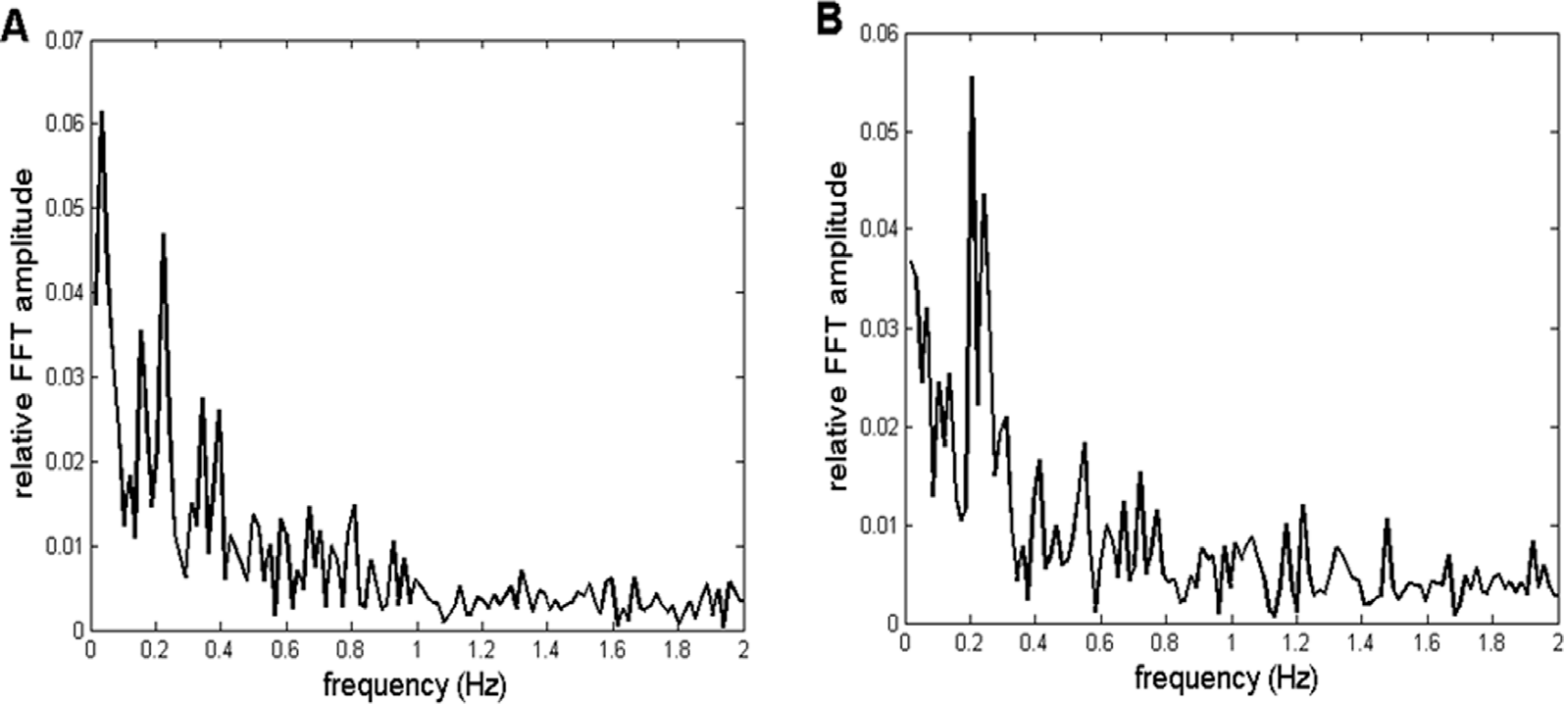
Two representative examples of relative amplitudes of *CFPS*(*t*) FFT spectra. Profiles are similar, exhibiting different individual oscillations superimposed on an fGn + fBm type noisy background. **(A)** Channel pair F8, T4 recorded in subject 7. **(B)** Channel pair F8, O2 is recorded in subject 9.

In this work, we tried to validate our method by subjecting an artificial, mathematically generated signal to the same procedure as the recorded *CFPS*(*t*) time dependencies. To achieve this, we first simulated the profiles of relative FFT amplitudes of typical *CFPS*(*t*) spectra. Two of them are presented in Figure 3.

As can be noticed, spectra are complex, consisting of different individual oscillations superimposed on a noisy background. However, these individual peaks did not appear in all 910 spectra. Specifically, they were more visible in spectra of some of the channel pairs than those in others, while altogether missing in some. In addition, inter-individual variability prevented them from appearing in all subjects for a given pair of EEG channels. Despite this complex behavior, in order to validate the method, it is necessary to perform the following five steps:

a) Generate an appropriate simulated background (of the corresponding fractal nature), as well as one individual artificial oscillation, preferably in the frequency range 0 < f < 0.5 Hz (according to the profiles shown in Figure 3), and add them mathematically.
b) Generate two synthetic 10-Hz carrier frequency “alpha” signals, with arbitrary constant (or random) amplitudes (Bojić et al., 2010).
c) Perform phase modulation of one of the signals in b) with the signal described in a).
d) Perform phase demodulation, *i.e.*, calculate *CFPS*(*t*) (according to the procedure described in our previous works, Kalauzi et al., 1998; Kalauzi et al., 2012b) between the modulated and unmodulated synthetic “alpha” signals, perform the ARE procedure, and calculate the FFT spectrum of the resulting *CFPS*(*t*) time series.
e) Compare and statistically test the original signal in a) against the demodulated *CFPS*(*t*), as well as their FFT spectra.

##### 2.2.4.1 Simulating the *CFPS*(*t*) background activity

In order to identify to which type of signals, such as two *CFPS*(*t*) examples shown in Figure 1, belong to, we reverted to the well-known but simple criteria explained in some previous papers (Eke et al., 2000) and employed in some of our previous works (Bojić et al., 2010), where the value of the exponent *β* determines its fractal nature. This exponent is given by the linear regression slope of the signals’ power vs. the frequency log–log plot:

$${\left|A\left(f\right)\right|}^{2}\propto cf^{-\beta },$$ (7)

where *c* stands for the proportionality factor. According to this classification, the critical value of *β* is 1 since for −1 < *β* < 1, the signal is identified as the fractal Gaussian noise (fGn), which is a stationary signal with constant variance. On the other hand, if 1 < *β* < 3, the time series is regarded as the non-stationary fractal Brown motion (fBm), in which signal variance increases with its length:

$$var\left(X\left(t\right)\right)\propto t^{2H},$$

where *X* denotes the current signal value and *H* is the Hurst exponent (Mandelbrot and van Ness, 1968; Eke et al., 2000).

**FIGURE 4:**
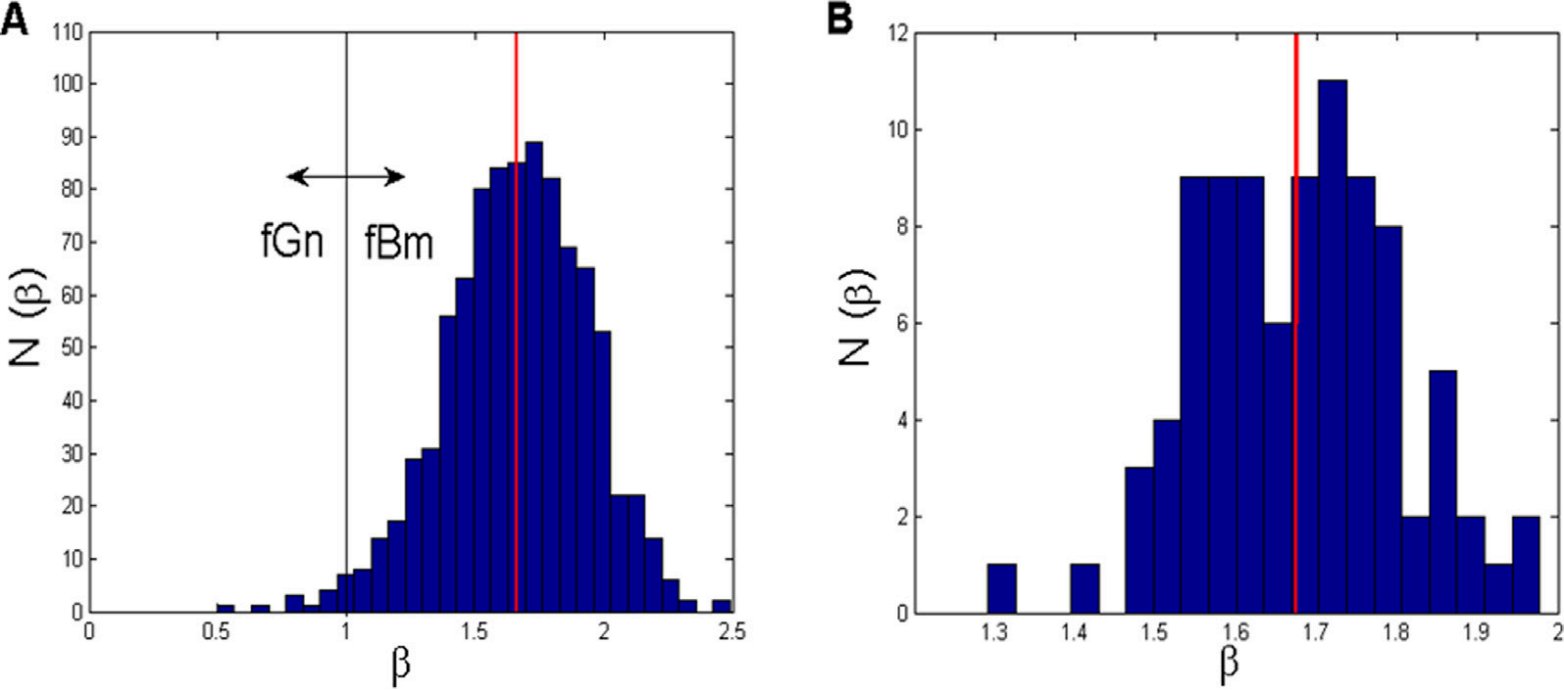
**(A)** Histogram of all 910 *β*-values (91 channel pairs × 10 subjects) calculated according to Equation 7. **(B)** Histogram of 91 *β*-values calculated for each channel pair by averaging the values across the group of 10 subjects, according to Equation 8. Vertical red lines mark the mean *β*.

As we could not find any specific data about the fractal nature of *CFPS*(*t*) in the literature, we performed linear regressions of all 1820 log–log power vs. frequency *CFPS*(*t*) signals (91 channel pairs × 10 subjects × 2 states) and histogrammized the corresponding beta values. The results are presented in Figure 4.

Let $\beta_{s,st}^{c1,c2}$ denote this quantity between channels *c1* and *c2* of subject *s* in state *st* = {wake, drowsy}, which was obtained from *CFPS*(*t*) using Equation 7. As shown in Figure 4A, all 910 such values were histogrammized, showing a unimodal distribution around mean(*β*) = 1.6596 ± 0.2741. Interestingly, the majority (98.57%) of the values were 1 < *β* < 2.5 and could be characterized as fBm, with only 13 (1.43%) belonging to the fGn type (*β* < 1). However, we average individual beta values for each channel pair across the tested group and denote them as

$$\beta_{st}^{c1,c2}=\frac{1}{10}\sum_{s=1}^{10}\beta_{s,st}^{c1,c2}.$$ (8)

The corresponding histogram (Figure 4B) points to a bimodal distribution: one mode localized at approximately *β*_*1*_ = 1.59 and the other at approximately *β*_*2*_ = 1.72. In this case, the overall mean is (*β*) = 1.6741 ± 0.1242. However, all these *β*-values, although not originating from individual signals but were a result of averaging, had an “fBm nature” (1 < *β* < 2).

There are different ways to generate a synthetic signal with desired fractal properties, such as using Weierstrass functions (Esteller et al., 2001; Kalauzi et al., 2005), in which the signal fractal dimension (*D*) is theoretically given and directly linked to one of the two parameters (*γ*, *H*):

$$W_{H}^{\gamma }\left(t\right)=\sum_{t}\gamma^{-iH}\cos\;\left(2\pi \gamma^{i}t\right),$$

where 0 < H < 1, γ > 1, and *H* = 2 - *D*.

**FIGURE 5:**
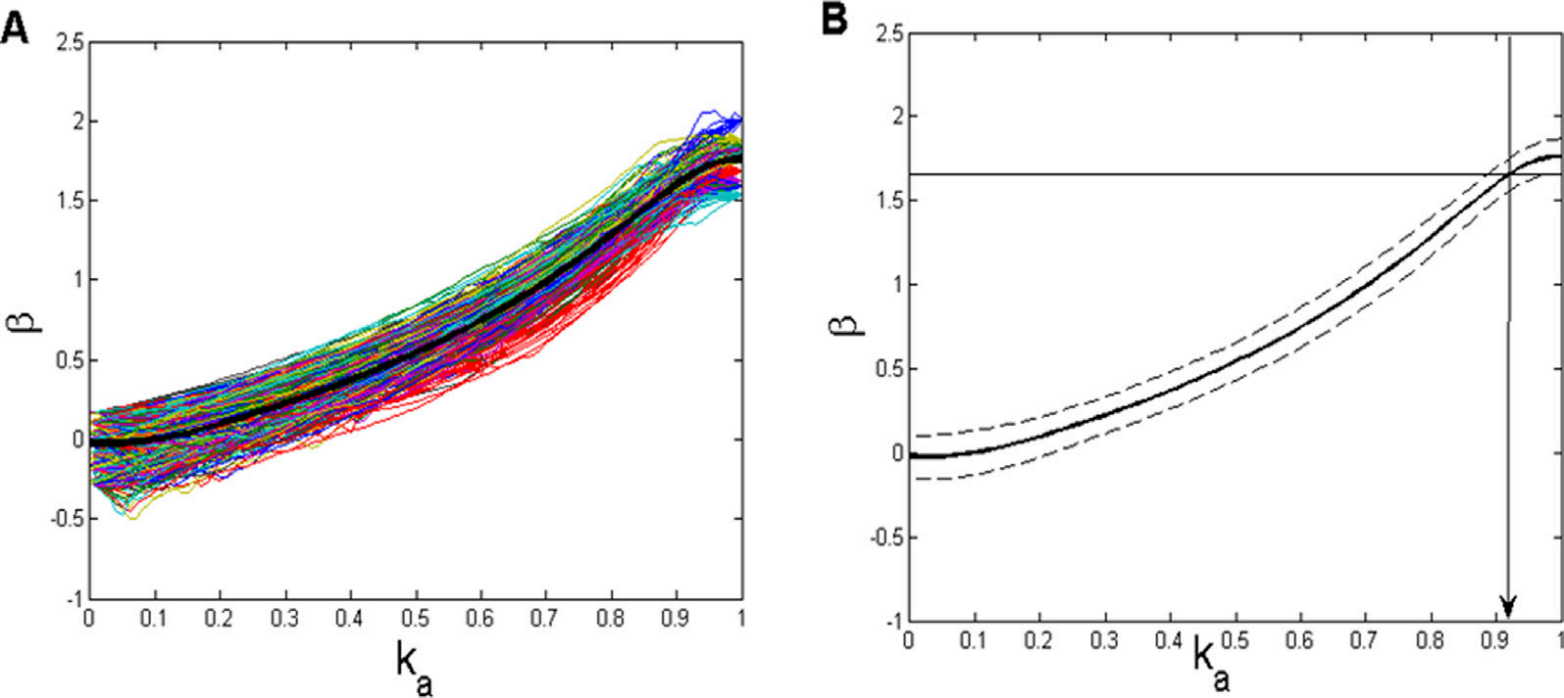
**(A)** Dependence of exponent *β* (Equation 7) on the relative amplitude attenuation factor *k*_*a*_ (Equation 9) for 20 repeatedly generated white noise and 20 Brownian motion signals. Thin lines represent 400 of these repetitions, whereas the thick black line represents their average. **(B)** Average (solid) ± standard deviation (dashed) of the same set of lines as shown in panel **(A)**. Mean *β* (1.6596), from the histogram shown in Figure 4A, is plotted as a thin horizontal line in order to determine the corresponding *k*_*a*_ value (≈0.92) to be used in the *CFPS*(*t*) background simulation.

However, the range of *β*, found in the histogram abscissa of Figure 4A, suggests that both fGn and fBm contribute to the FFT background of *CFPS*(*t*) signals. This circumstance may point to an alternative way of generating a signal with the desired *β*-value—a composite fGn/fBm. This fractal signal can be obtained by summing the two componential signals with various relative amplitude contributions. For this purpose, we used the approach described in Kalauzi et al. (2012a), where two signals with inverse amplitudes were summed while keeping the amplitude of the sum constant:

$$A_{fGnfBm}=\left(1-k_{a}\right)A_{fGn}+k_{a}A_{fBm},$$ (9)

where *A*_*fGnfBm*_ stands for the amplitude of the composite fGn + fBm signal, *A*_*fGn*_ and *A*_*fBm*_ denote amplitudes of the two componential signals, and *k*_*a*_ is the relative amplitude attenuation factor (0 ≤ *k*_*a*_ ≤ 1). A custom MATLAB program was made to calculate how *β* of the composite fGn + fBm signal depends on *k*_*a*_. The result is presented in Figure 5. In order to cover the whole beta range (0 < *β* < 2), fGn was represented by a synthetic white noise Wn(*i*), *i* = 1,.,233, acting as a random variable with the uniform probability distribution U (−0.5, 0.5). Then, we derived the Brown motion signal, Bm(*j*), *j* = 1,.,233, representing the fBm component, by numerically summing white noise samples: $Bm\left(j\right)=\sum_{i=1}^{j}Wn\left(i\right)$ , *j* = 1,.,233.

Subsequently, we aimed to establish a *β* = f (*k*_*a*_) dependence, from which, using it as a sort of calibration line, the value of *k*_*a*_, corresponding to the mean *β*-value, obtained from the 910 *CFPS*(*t*) signals (mean(*β*) = 1.6596; Figure 4A), could be determined. Since both Wn and Bm signals, generated with a relatively small number of samples in order to match the recorded signals, might show some statistical variability, we repeated the construction of the *β* = f(*k*_*a*_) calibration line using 20 Wn and 20 Bm signals, obtaining a field of 400 combination lines (Figure 5A). In addition, since these lines also depend on the initial amplitudes of both Wn and Bm components, amplitude normalization had to be carried out:

$$y_{N}\left(i\right)=\frac{y\left(i\right)-mean\left(y\right)}{std\left(y\right)},$$

where *y*(*i*) stands for the *i*th sample value of either of the fractal signals (Wn or Bm).

**FIGURE 6:**
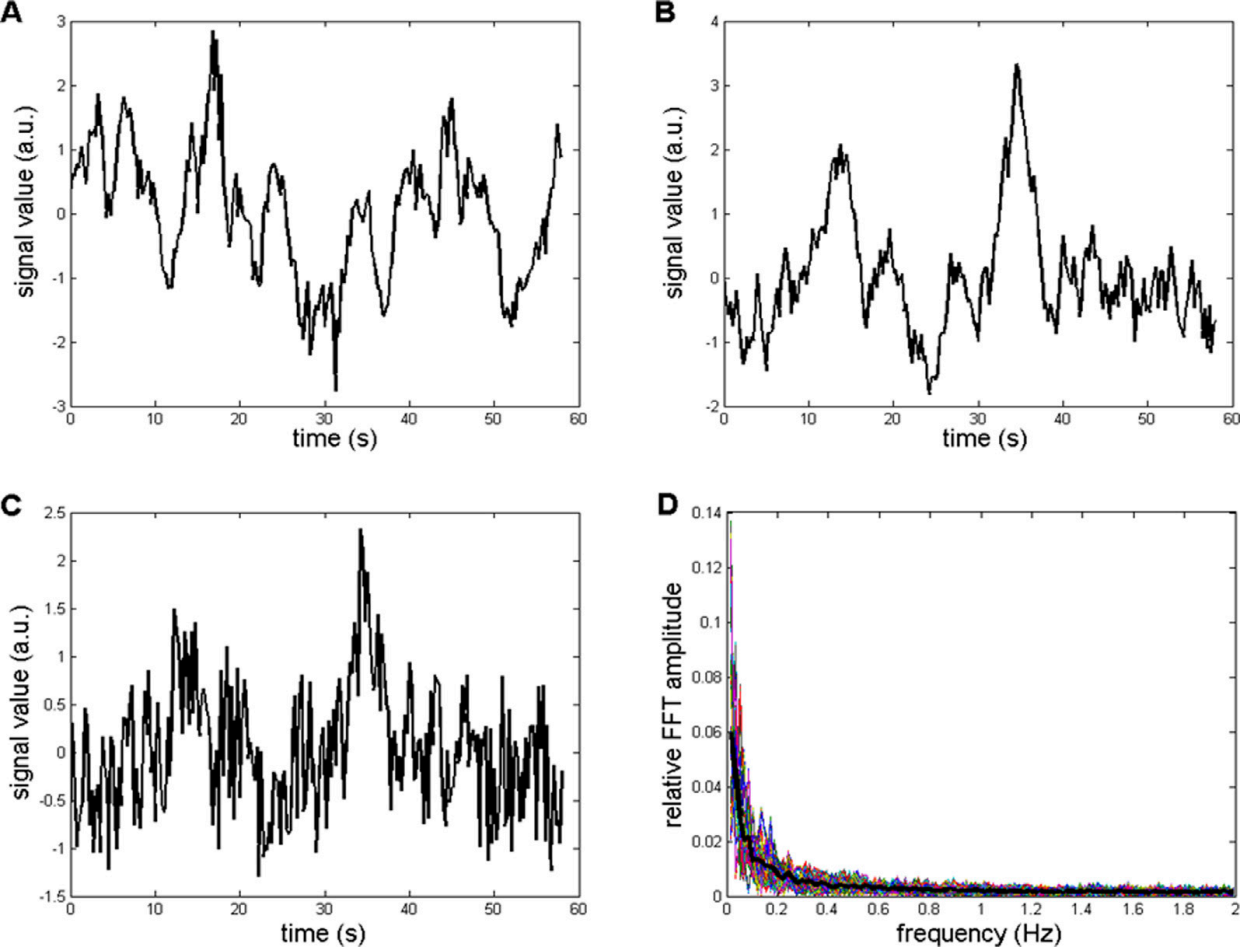
Comparison between recorded and simulated *CFPS*(*t*) background activities in the time domain. **(A)** Recorded from subject 1: channels F8, T4. **(B)** Simulation containing Wn and Bm, with *k*_*a*_ = 0.92. **(C)** Simulation with *k*_*a*_ = 0.5. **(D)** Relative amplitude FFT spectra of 400 repeated simulations generated using *k*_*a*_ = 0.92. Thin colored lines represent individual simulations, whereas the thick black line represents their average.

The importance of precisely determining the coefficient *k*_*a*_ for generating a successful background simulation is illustrated in Figure 6. Figure 6A shows an example of the recorded *CFPS*(*t*) (subject 1; EEG channels F8, T4), while Figures 6B, C contain simulated background activity for *k*_*a*_ = 0.92 and *k*_*a*_ = 0.5, respectively. The similarity of signals shown in Figures 6A, B is obvious, which is contrary to Figures 6A, C.

**FIGURE 7:**
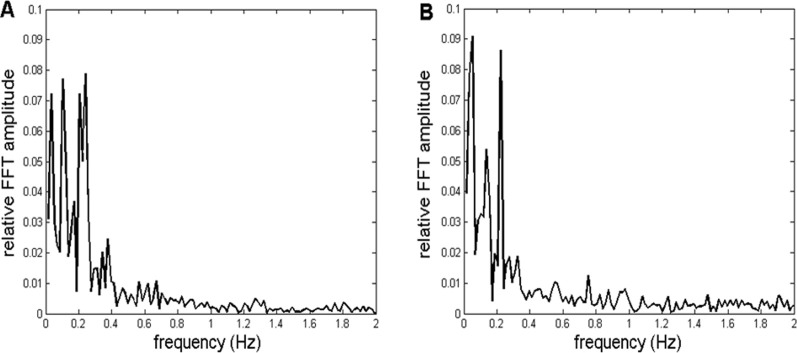
**(A)** Representative example of a relative amplitude spectrum of recorded *CFPS*(*t*) activity, with pronounced individual oscillations superimposed on the background, obtained from subject 9, between channels O1 and O2. **(B)** Simulation of the activity shown in **A**, consisting of a fGn + fBm (represented by Wn + Bm) fractal background, with *k*_*a*_ = 0.92 and summed with a 0.22-Hz artificial sinewave.

Finally, to the background presented in Figure 6B, a mathematically synthesized sinusoid of 0.22 Hz frequency was added in order to simulate the situation where both the background and the most prominent recorded oscillation was present (such as those shown in Figures 3, 7A). Relative FFT spectra of the simulated, as well as an example of one of the recorded signals, are shown in Figure 7. It is important to note that since the recorded *CFPS*(*t*) signals differed among themselves, it was not necessary to simulate any of them exactly; for method validation, it was sufficient that the simulation reproduced signal’s main properties.

**FIGURE 8:**
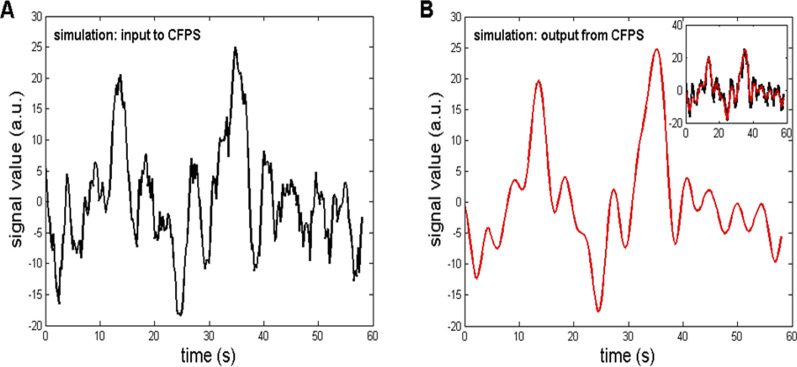
**(A)** Simulation of a typical *CFPS*(*t*) waveform, containing two fractal background components, Wn and Bm, and a 0.22-Hz sinewave. The simulation served as the input to the procedure of calculating *CFPS*(*t*), which is part of the steps a)–e) in Section 2.4. **(B)** Output from the *CFPS* procedure, having a high correlation with the input (r = 0.9421). **Inlet**: input–output waveforms superimposed.

Once generated, the simulated signal was used to follow the procedures described in steps a)–e) in Section 2.4 and also used as the input to the *CFPS*(*t*) calculation described in detail in our previous papers (Kalauzi et al., 1998; Kalauzi et al., 2009; Kalauzi et al., 2012b) and in this study in Section 2.2. For the method to be validated, the input signal should resemble, as much as possible, the output signal obtained after completing the abovementioned steps. Their comparison is shown in Figure 8. The coefficient of linear correlation between the input and output signals was *r* = 0.9421, and it was highly significant (*p* = 1.46 × 10^–111^). The resulting (output) signal shows (Figures 8A, B) that some higher frequency oscillations were attenuated due to the low-pass filtering effect of the 2-s moving Fourier window (Kalauzi et al., 2023a). However, this attenuation has little effect on our main finding regarding the presence of *CFPS*(*t*) oscillations because they are positioned at ≈ 0.22 Hz, while the first filter zero is at 0.5 Hz. Moreover, this attenuation is equal for all 91 channel pairs so that *CFPS*(*t*) waveforms and their FFT amplitudes could be directly compared.

If we denote with $A_{s,st}^{c1,c2}\left(f\right)$ the FFT amplitude spectra of *CFPS*(*t*) waveforms between channels *c1* and *c2*, recorded in subject *s* in state *st* (analogously to the notation of Equation 8), and average them across the tested group and all channel pairs in order to get one resulting spectrum, we obtain

$$\bar{\bar{{\left(A_{r}\right)}_{st}\left(f\right)}}=\frac{1}{910}\sum_{c1,c2=1}^{91}\sum_{s=1}^{10}{\left(A_{r}\right)}_{s,st}^{c1,c2}\left(f\right),$$ (10)

where *A*_*r*_ stands for the relative amplitude:

$$A_{r}\left(f\right)=\frac{A\left(f\right)}{\sum_{f}A\left(f\right)}.$$

Finally, spectra averaging across the group for each channel pair result in the following expression:

$$\bar{{\left(A_{r}\right)}_{st}^{c1,c2}\left(f\right)}=\frac{1}{10}\sum_{s=1}^{10}{\left(A_{r}\right)}_{s,st}^{c1,c2}\left(f\right).$$ (11)

A custom MATLAB program was made to select the given number of channel pairs with spectra having the maximal sum of relative amplitudes in the chosen frequency region.

## 3 Results

### 3.1 Results of FFT analysis applied on ARE-corrected alpha *CFPS*(*t*) waveforms of a test group in the wake state

**FIGURE 9:**
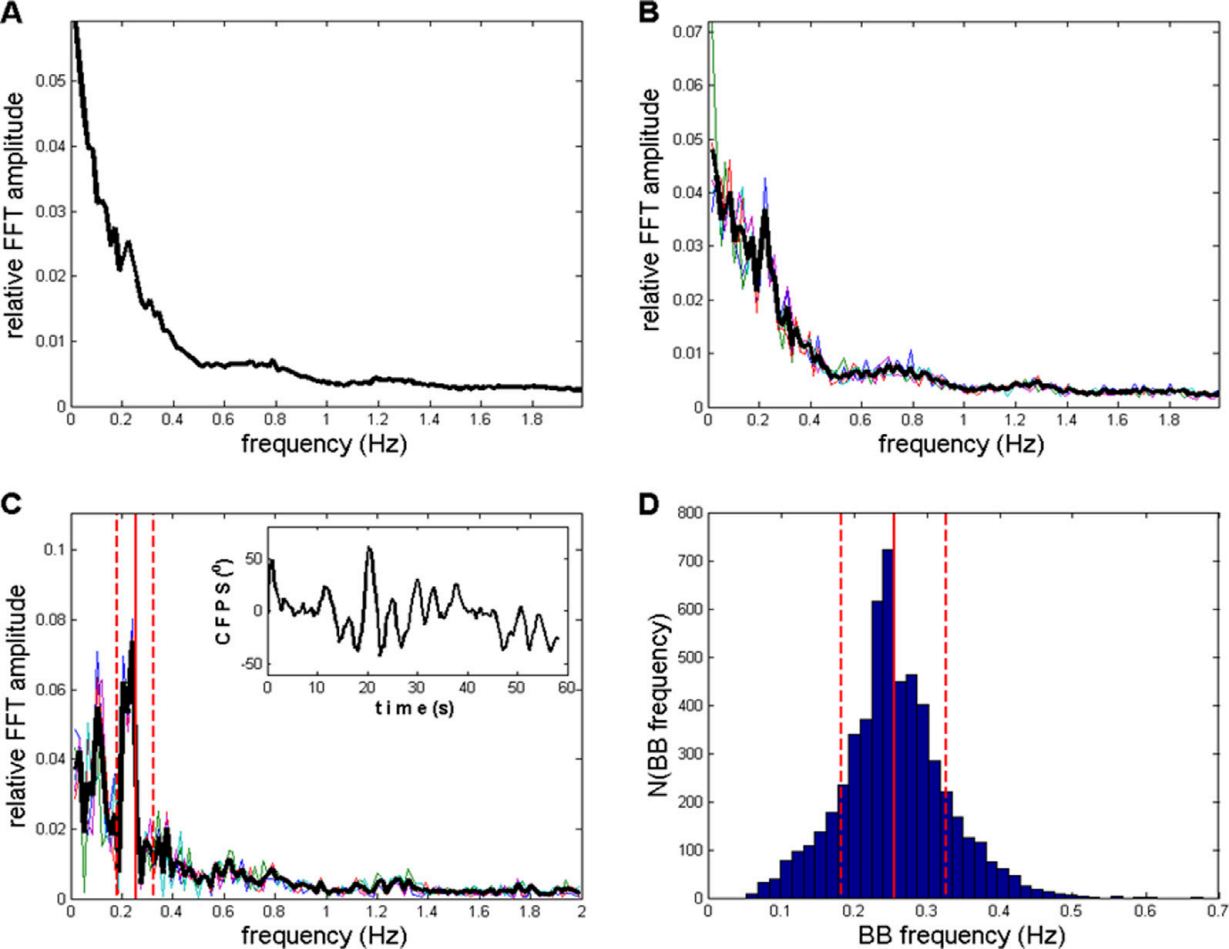
**(A)** Average of all 910 (10 subjects x 91 channel pairs) FFT *CFPS*(*t*) spectra according to Equation 10; no selection applied. A peak at the 13th FC (0.2232 Hz) is small but visible. **(B)** Average of five spectra, previously averaged across 10 subjects (Equation 11), selected to have maximal sum of relative amplitudes between 12th and 15th FCs (0.2060–0.2575 Hz). The same peak appeared, with an increased S/N ratio compared to the ratio in panel **(A)**. **(C)** Average of five spectra, with no previous group averaging (i.e., directly from the pool of 910 spectra), again characterized by the maximal sum of relative amplitudes between 12th and 15th FCs. Main peaks were in the range of 0.2060–0.2403 Hz, as well as one appearing at sixth FC (0.1030 Hz). **Inlet**: an example of recorded *CFPS*(*t*), subject 9, channels O1 and O2, where respiratory frequency oscillation could be noticed by the naked eye. **(D)** Histogram of 5450 BB (breath-to-breath) frequencies recorded from 20 healthy subjects in the supine position. Vertical red lines, with mean ± st. dev., also drawn in panel **(C)**. Thin colored lines in panels **(B, C)** originate from five individual spectra, whereas the thick black line represents their average.

**FIGURE 10:**
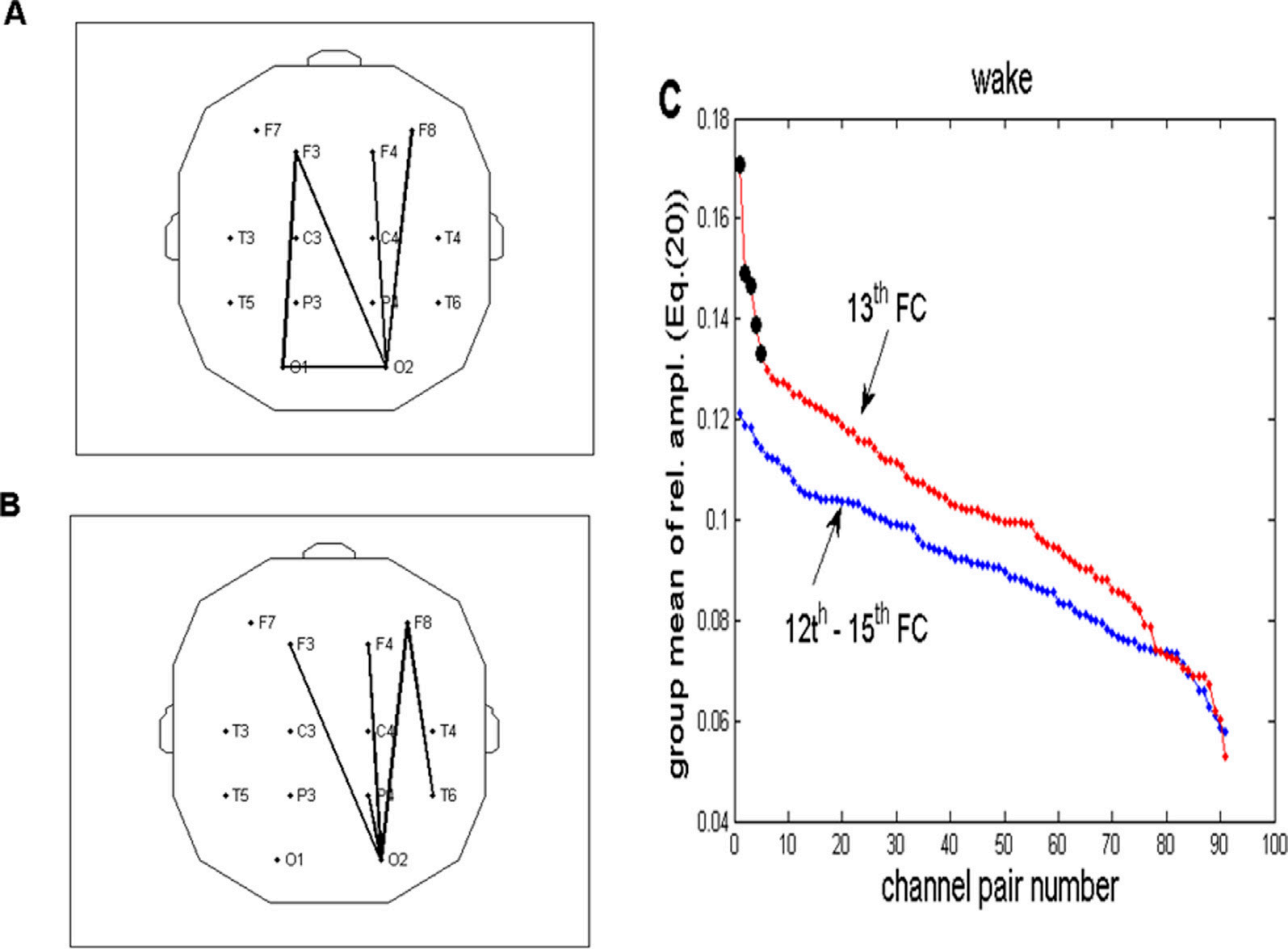
Topographic view of the selection of five channel pairs with a maximal group average of relative FFT amplitudes in the wake state, according to Equation 11. **(A)** Channel pairs with the maximal sum of four relative amplitudes (12th–15th FCs), corresponding to the frequency range of 0.2060–0.2575 Hz. **(B)** Analogous topography shown in panel **A** drawn for one (13th FC) at a frequency of 0.2232 Hz. **(C)** Relative FFT amplitudes for all 91 channel pairs (Equation 11) plotted in the descending order. Ordinates of the red line (representing one FC) were taken from Table 2 and multiplied by four to make them comparable to the blue line (corresponding to summed amplitudes of 4 FCs). The first five channel pairs of the red line are marked with black circles because they correspond to the pairs plotted in panel **(B)**. Their abrupt decrease explains why we chose five pairs for the topographic presentation. For the sake of clarity, error bars were omitted.

The spectrum defined in Equation 10 is presented in Figure 9A. The profile of this spectrum shows some particular oscillations superimposed on the formerly analyzed background activity, consisting of mixed fGn and fBm fractal signals, with the corresponding *β* exponents’ histogram presented in Figure 4A. Amplitude variability visible at f > 0.5 Hz belongs to sidelobes, which result from the low-pass filtering effect of the 2-s FFT moving window. The most visible individual peak is the one positioned at 0.2232 Hz (13th FC). However, individual spectra (from the pool of 910 available) differ among each other in amplitudes and exact positions of these peaks, which appear in the 0 < f < 0.5 Hz region. In order to explore which channel pairs exhibit most prominent relative FFT amplitudes on *f* = 0.2232 Hz and hopefully increase the signal-to-noise ratio (S/N), the first step was to perform spectra averaging across the group for each channel pair according to Equation 11. If we choose to average and present five channel pairing with the maximal sum of relative amplitudes in the region of 12th–15th FCs (0.2060–0.2575 Hz), according to Equation 11, the resulting spectrum is presented in Figure 9B, where the improved S/N ratio is apparent compared to that shown in Figure 9A. The list of these five channel pairs, as well as the corresponding values of $\bar{{\left(A_{r}\right)}_{w}^{c1,c2}\left(0.2060\;–\;0.2575\right)}$ , is listed in Table 2, while the topographic distribution of these channel pairs is plotted in Figure 10A, where mostly longitudinal (fronto-occipital) channels are connected. Analogous calculation was carried out for one FC (13th) by applying $\bar{{\left(A_{r}\right)}_{w}^{c1,c2}\left(0.2232\right)}$ . The result is also given in Table 2 and shown in Figure 10B. As can be seen, pairs of connected EEG channels were similar in both cases.

**TABLE 2:** Upper part: Five channel pairs with maximal sum of 12th–15th FC relative FFT amplitudes and their corresponding $\bar{{\left(A_{r}\right)}_{st}^{c1,c2}\left(f\right)}$ values (group mean ± st. dev.). Lower part: The same calculation for only one (13th) FC.

Wake		Drowsy	
Channel	$\bar{{\left(A_{r}\right)}_{w}^{c1,c2}\left(0.2060\;–\;0.2575\right)}$	Channel	$\bar{{\left(A_{r}\right)}_{d}^{c1,c2}\left(0.2060\;–\;0.2575\right)}$
F8, 02	0.1214 ± 0.0396	F8, F4	0.0913 ± 0.0249
F4, 02	0.1187 ± 0.0486	C3, 02	0.0911 ± 0.0223
01, 02	0.1185 ± 0.0503	01, 02	0.0898 ± 0.0372
F3, 01	0.1154 ± 0.0441	C4, P4	0.0872 ± 0.0300
F3, 02	0.1144 ± 0.0354	T5, 01	0.0850 ± 0.0156

Channel	$\bar{{\left(A_{r}\right)}_{w}^{c1,c2}\left(0.2232\right)}$	Channel	$\bar{{\left(A_{r}\right)}_{d}^{c1,c2}\left(0.2232\right)}$
F8, 02	0.0427 ± 0.0123	F8, F4	0.0285 ± 0.0132
F8, T6	0.0373 ± 0.0194	C3, 02	0.0280 ± 0.0091
F4, 02	0.0367 ± 0.0170	01, 02	0.0279 ± 0.0145
F3, 02	0.0348 ± 0.0157	C4, P4	0.0257 ± 0.0106
P4, 02	0.0332 ± 0.0140	F8, F3	0.0252 ± 0.0131

Finally, if we want to eliminate the influence of inter-individual variability on oscillation detection and appearance, and perhaps even further improve the S/N ratio, no primary averaging should be performed, and five spectra with the maximum sum of relative amplitudes in the 0.2060–0.2575 Hz region should be selected directly from the pool of all 910 available spectra. The resulting average of these five finally selected spectra is presented in Figure 9C. In this case, main peaks were in the range of 0.2060–0.2403 Hz; however, a peak at the sixth FC (0.1030 Hz) also appeared. A further increase in the S/N ratio was herewith achieved, with even less fractal-type background activity present. In order to identify, not conclusively but to a reasonable degree of probability, the possible physiological origin of the oscillation positioned at ≈ 0.21–0.24 Hz, we used breath-to-breath (BB) interval data from our previous experiments (Matić et al., 2020; Matić et al., 2022; Kalauzi et al., 2023b), where respiratory signals from 20 healthy subjects in the supine position were recorded. A histogram of their 5,450 pooled instantaneous breathing frequencies (*B*_*f*_), which were obtained as the inverse of the corresponding BB intervals, is presented in Figure 9D. These pooled data had a mean (*B*_*f*_) = 0.2552 ± 0.0715 Hz and are indicated as vertical red lines in Figures 9C, D. From this comparison, it can be seen that the position of the ≈0.21–0.24 Hz FFT *CFPS*(*t*) peak lies within the expected frequency boundaries of relaxed healthy human breathing, although slightly shifted toward lower frequencies. If the breathing origin hypothesis is adopted, the small shift could be explained by the fact that the 10 EEG subjects were more relaxed than the 20 respiratory signal subjects (Matić et al., 2020; Matić et al., 2022) as the former were in the wake state but not far from the drowsy state (Vuckovic et al., 2002; Kalauzi et al., 2012b). Final confirmation of the respiratory connection of detected *CFPS*(*t*) oscillations is expected to be obtained by simultaneously recording both respiratory and EEG signals from a sufficient number of subjects, preferably in different cardio-respiratory conditions, where frequency matching and phase locking between respiratory and *CFPS(t*) oscillations can be accurately established.

In addition to topographic distributions on Figures 10A‐C, show group averages of relative FFT amplitudes for all 91 channel pairs, arranged in descending order using Equation 11. Ordinates of the red line (referring to 1 FC) were derived from Table 2 and multiplied by 4 to make them comparable to the blue line, which depicts the group average of four summed FC (12th–15th) amplitudes. Notably, first five channel pairs of the red line are marked with black circles because they correspond to the pairs plotted in Figure 10B. Five pairs were chosen because they belong to the part of the line where an abrupt decrease occurs.

### 3.2 Results of FFT analysis applied on ARE-corrected alpha *CFPS*(*t*) waveforms of a test group in the drowsy state

The same procedure was applied to the recorded signals from the same 10 subjects in the drowsy and wake states, allowing the results to be directly compared.

**FIGURE 11:**
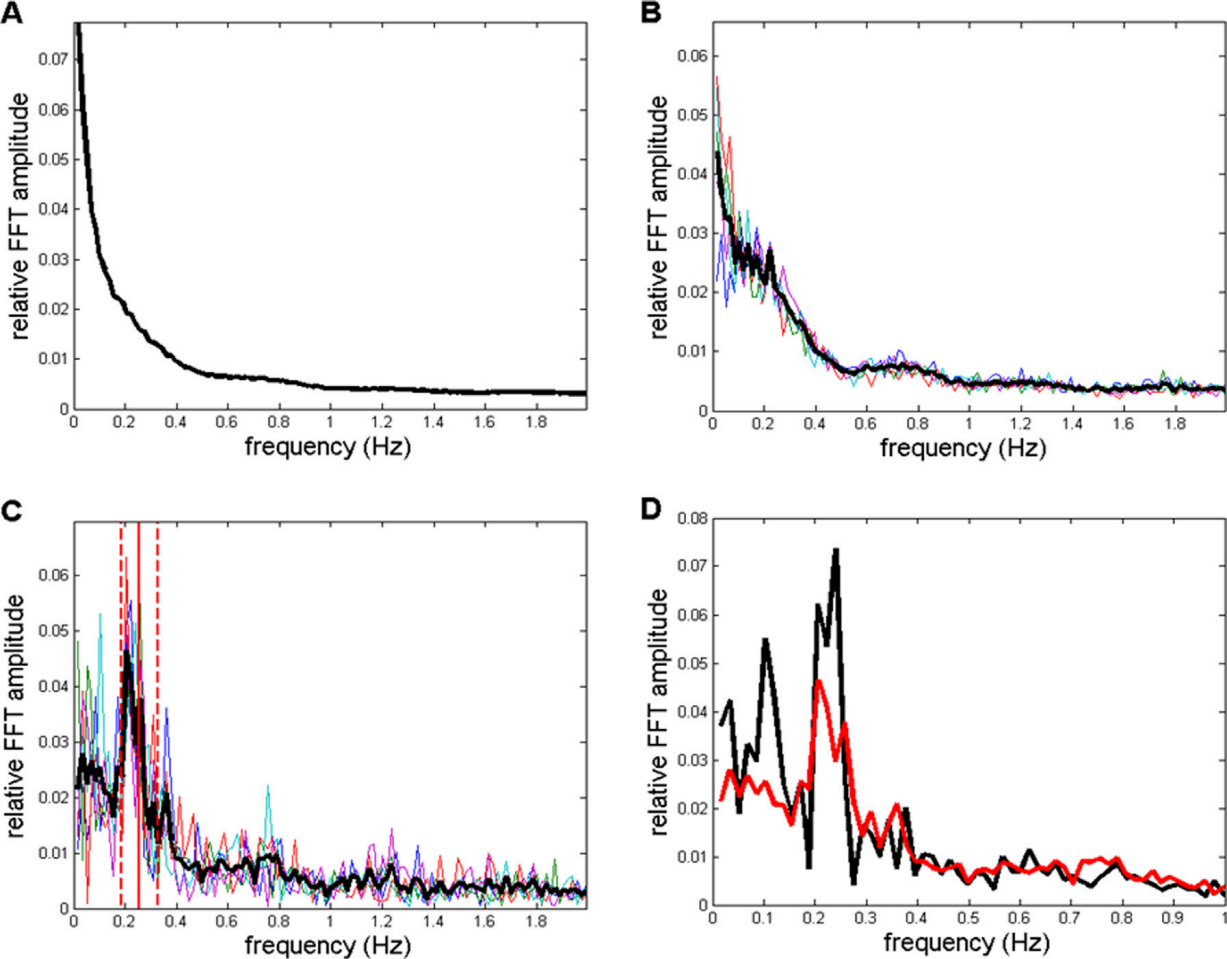
**(A**–**C)** Spectra corresponding to those shown in Figures 9A–C, but here, they refer to the drowsy state. **(A)** *CFPS* oscillation peaks in the low-frequency region f < 0.5 Hz are reduced or even diminished compared to the wake state, as shown in Figure 9A. **(B)** After averaging across the 10 subjects, in five selected spectra, a reduced 13th FC (0.2232 Hz) peak appeared in the drowsy state as well. **(C)** The sixth FC peak at 0.1030 Hz, which was present in the wake state shown in Figure 9C, disappeared in the drowsy state. **(D)** Averaged spectra from Figures 9C, 11C are superimposed so that they can be directly compared.

**FIGURE 12:**
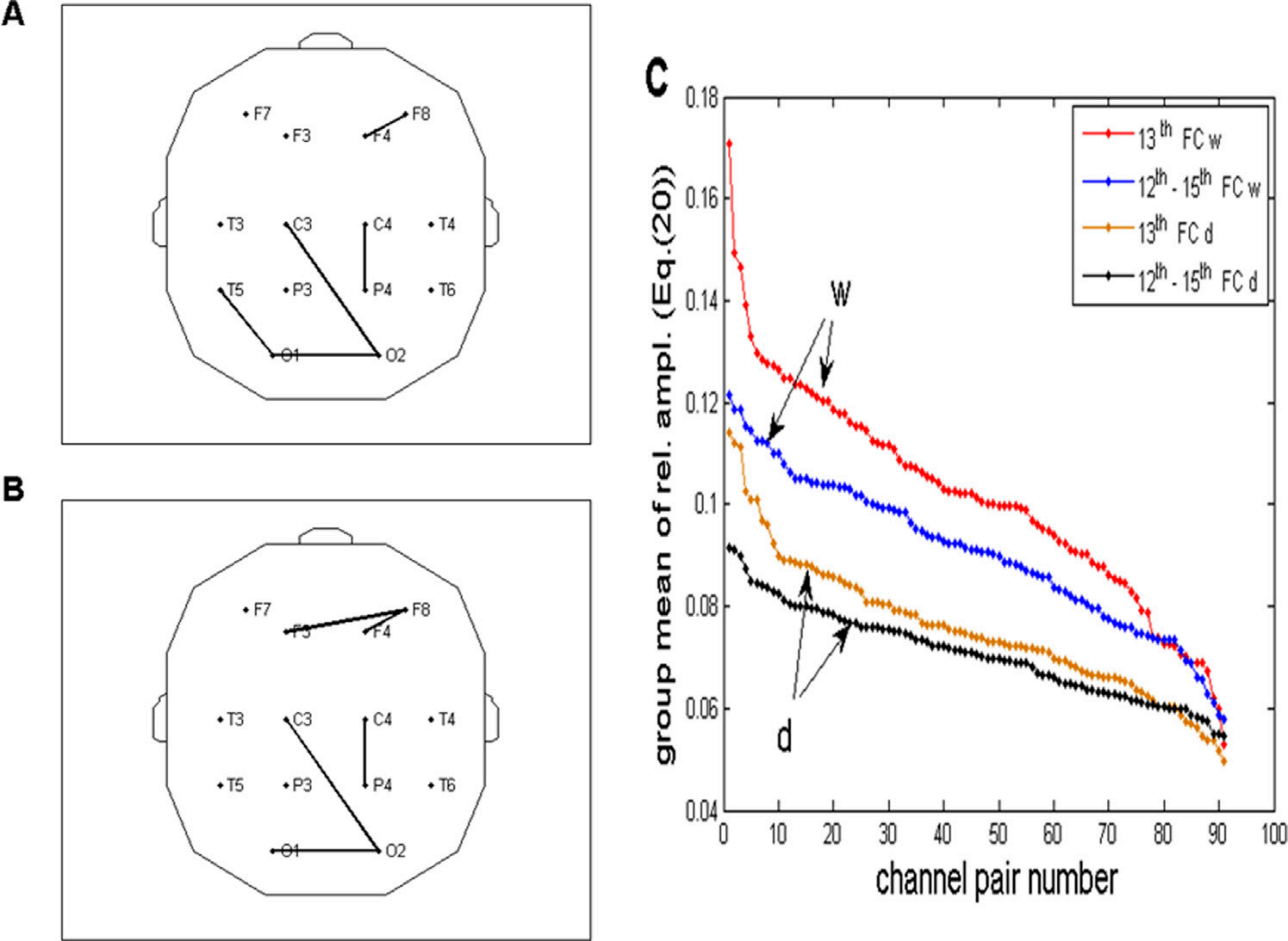
**(A, B)** Same topographic distribution of five channel pairs is shown in Figures 10A, B, obtained for the drowsy state. As shown in the wake state, the two distributions are mutually similar but differ significantly from the distributions in the wake state. **(C)** Relative FFT amplitudes for all 91 channel pairs (Equation 11) plotted in the descending order, superimposed for the wake (w) and drowsy (d) states and for two frequency regions (13th and 12th – 15th FCs). As shown in Figure 10C, ordinates of the brown line (one FC) were multiplied by four to be comparable to the black line (summed amplitudes of 4 FCs). For all channel pairs, amplitudes in the drowsy state are smaller than those in the wake state, pointing to the fact that wakefulness is necessary for the respiratory frequency alpha *CFPS* to be present in full capacity across the whole scalp.

Drowsy state results are displayed in Figures 11, 12. Figures 11A–C essentially show the same information as that presented in Figures 9A–C. Figure 11D shows a direct comparison of averaged spectra from those shown in Figures 9C, 11C. From all panels, as shown in Figure 11, one can observe a reduction in individual peaks in the low frequency region (f < 0.3 Hz) compared to the wake peaks shown in Figure 9.

The topographic distribution of channel pairs, characterized by five greatest group averages of summed four relative FFT amplitudes in the range of 0.2060–0.2575 Hz (12th to 15th FCs; Figure 12A), or one FC positioned at 0.2232 Hz (13th FC; Figure 12B), was mutually similar but differed significantly from the two distributions obtained in the wake state (Figures 10A, B). Specifically, pairs connecting fronto-occipital regions, recorded in the wake state, did not emerge in the drowsy state.

### 3.3 Statistical testing of relative amplitude differences between the wake and drowsy states

**FIGURE 13:**
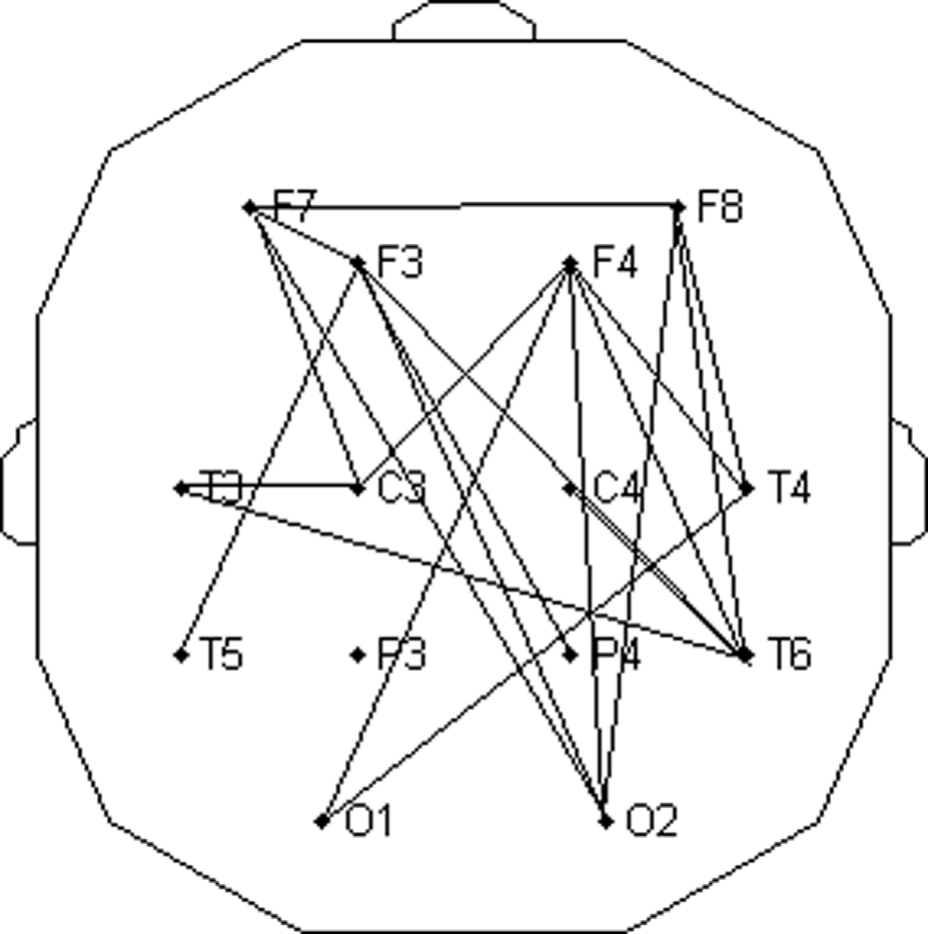
Topographic distribution of channel pairs which had significantly greater (*p* < 0.05) relative FFT amplitudes of *CFPS*(*t*) oscillations at 0.2232 Hz in the wake state than those in the drowsy state.

The channel pairs shown in Figure 12C were not arranged and presented in the same order since each line reflected its own descending order, rendering them unsuitable for statistical testing. Therefore, we rearranged the order of channel pairs of the drowsy state (corresponding to the order on the brown line shown in Figure 12C) to match the descending wake order (corresponding to the red line on the same panel). Using the Wilcoxon matched-pairs test, we tested differences between wake and drowsy group ensembles of FFT (*CFPS*(*t*)) relative amplitudes at 0.2232 Hz (13th FC) for each of the 91 channel pairs. Therefore, each of the two input ensembles for the 91 tests consisted of 10 values, one for each subject, defined in Section 3.1 as ${\left(A_{r}\right)}_{s,w}^{c1,c2}\left(f\right)$ and ${\left(A_{r}\right)}_{s,d}^{c1,c2}\left(f\right)$ , with *s* = 1,.,10. Here, *c*1 and *c*2 stand for the two EEG channels, while *w* and *d* subscripts denote wake and drowsy states, respectively. Of the 91 tested pairs, 20 appeared to have significantly greater (*p* < 0.05) amplitudes in the wake state. All group ensemble values, averages of relative amplitudes in wake and drowsy states, and the corresponding *p*-values can be obtained upon request from the authors. In this study, we shall present only five pairs with the most significant differences (*p* < 0.01): F402 and T4F4, *p* = 0.0051; T3T6 and F7F8, *p* = 0.0069; and F8T6, *p* = 0.0093. The topographic distribution of all 20 pairs is presented in Figure 13. As can be seen, diagonal directions were preferred, with more lines connecting left anterior to right posterior scalp regions than those in the opposite direction.

## 4 Discussion

In this work, the carrier frequency mathematical model of human EEG alpha oscillations was applied to 10 young healthy participants in wake and drowsy states to calculate the time course of *CF* phase shifts *CFPS*(*t*) for all 91 channel pairs. After the validation of the method, their oscillatory content was studied through FFT (*CFPS*(*t*)). Although not appearing equally in all channel pairs and every subject, a clear peak in the respiratory frequency region, 0.21–0.26 Hz, was observed (max at ≈ 0.22 Hz). When five channel pairs with the most prominent group averaged amplitudes at 0.22 Hz were plotted in both states, topographic distributions changed significantly—from longitudinal, connecting frontal and posterior channels in the wake state to topographically split two separate regions—frontal and posterior in the drowsy state. Moreover, in the drowsy state, 0.22-Hz amplitudes decreased for all pairs, while statistically significant reduction was obtained for 20/91 (22%) pairs.

### 4.1 Alpha waves and neuronal excitability

Both the alpha amplitude and instantaneous phase influence neuronal excitability in the cortex. Sauseng et al. (2009) found that a motor-evoked potential was elicited more easily when alpha power immediately preceding the transcranial magnetic stimulation (TMS) pulse was low and *vice versa*. Similarly, Romei et al. (2008) reported that low pre-stimulus alpha-band power resulted in TMS reliably inducing positive responses, whereas high pre-stimulus values caused the same TMS stimuli to fail to evoke a visual percept during spontaneous fluctuations in posterior alpha activity. However, there are some contradicting results concerning the pre-stimulus amplitude (but not the phase). Berger et al. (2014) applied single-pulse transcranial magnetic stimulation (TMS) over the left sensorimotor cortex while simultaneously recording EEG. Results indicate that the instantaneous phase, but not amplitude, of oscillations in various frequency bands at the stimulation site during the time of TMS pulse correlates with different levels of excitability. The fact that neuronal excitability was dependent on the instantaneous alpha phase had been recognized for quite some time. Callaway and Yeager (1960) found that visual reaction time depended on different phases of the alpha cycle. More recently, Mathewson et al. (2009) showed that both subsequent visual detection and stimulus-elicited cortical activation levels in a metacontrast masking paradigm can be reliably predicted by the phase of alpha rhythm measured over posterior brain regions. When a target was presented during the trough (minimum) of an alpha wave, cortical activation was suppressed 100 m after the presentation, and participants were less likely to detect the target. They concluded that during one alpha cycle, the human brain goes through rapid oscillatory shifts in excitability, directly influencing whether an environmental stimulus will reach conscious awareness or not.

### 4.2 Respiration and neuronal excitability

On the other hand, respiration exerts its own impact on neuronal excitability. Neuronal activity phase-locked to respiration has been evidenced in many published results. Respiratory modulation of the power of gamma frequency oscillations was found in the whisker barrel cortex of wake mice (Ito et al., 2014; Heck et al., 2017). Jung F. et al. (2023) stated that “rhythmic body processes strongly influence activity patterns throughout the brain,” further mentioning respiration as a major factor. This influence is extended to both local field potential and single-cell levels. However, according to the authors, it is not known how respiratory-driven rhythms interact or compete with internal brain oscillations, especially if the frequency ranges overlap, such as respiratory and theta rhythms in mice. They report state-dependence of parietal cortex unit spike modulation by both rhythms: during REM sleep, theta dominated unit discharge, while respiration exerted greater influence during active waking. Heck et al. (2017) argued that “respiration, via multiple sensory pathways, contributes a rhythmic component to the ongoing cortical activity,” suggesting that “this rhythmic activity modulates the temporal organization of cortical neurodynamics, thereby linking higher cortical functions to the process of breathing.” Let us note that measurements of respiratory influence on the power of field potential oscillations or unit discharges, such as those in these three studies, differ in one important aspect from the approach involving phases, obtained by, e.g., Hsu et al. (2020) or ours, presented in this work. In other words, in the former case, each particular result originates from one static electrode, resulting in “static” results on the receiving end. In the phase (shift) approach, the results are “dynamic,” describing the interaction of phases of two oscillations: respiratory and field potential.

The combined results of these two lines of research indicate that in this work, we are dealing with two very complex interacting oscillatory systems, each one altering the excitability of extensive neuronal networks and affecting simultaneously all cortical areas. At this stage, when the phase–frequency interaction of the two excitability altering oscillations has only recently been detected, it is not easy to give a valid interpretation or give a meaningful proposal for either anatomical or physiological mechanisms that underlie the phenomenon.

However, some aspects of the issue can still be discussed even in this preliminary phase. Logically, minimal anatomical and consequently functionally active set of structures should include the PreBötzinger complex (preBötC) locus coeruleus and thalamo-cortical system. Influences between respiratory centers and the cerebral cortex are known to be bilateral. PreBötC projects respiratory afferents to cortical brain areas, e.g., via the locus coeruleus, from olfactory nuclei and through the vagus nerve (Del Negro et al., 2018; Kluger and Gross, 2020). In addition, the inspiratory rhythm originating from the preBötC activity coordinates associated conditional oscillators for other phases of the breathing cycle, for sighing and orofacial behaviors. Furthermore, the preBötC influences arousal, cognitive function, and emotion (Del Negro et al., 2018). In turn, brain states (such as anxiety) lead to changes in respiration. However, even if these structures and communication between them are included in the respiratory modulation of the alpha *CFPS*(*t*) oscillations, at this stage, it remains unclear through which particular mechanism could respiration, assuming our hypothesis is valid, be influencing the observed *CF* phase shifts.

In addition to neurons in the PreBötzinger complex, respiration is actively influenced by groups of neurons in the pons and medulla, as well as central and peripheral chemoreceptors, mechanoreceptors, and metaboreceptors. Pontine neuronal groups incorporate the pneumotaxic and apneustic centers, which participate in control of the speed of breathing and perform fine-tuning of the rate of respiration (Webster and Karan, 2020). Dorsal medullar neurons are responsible for inhalation and airway defense, while the ventral neurons control exhalation (Bolser et al., 2015; Pagliardini et al., 2011). However, although mutually well-connected and functionally well-organized, the main external connection of these medullar groups with cortical structures is achieved through the PreBötzinger complex and locus coeruleus (Del Negro et al., 2018; Webster and Karan, 2020). This fact allows us to further hypothesize that their role in the emergence of the detected *CFPS*(*t*) rhythm is indirect. In contrast, sensory receptors project to the breathing centers not the cortex itself (Berger and Averill, 1983; Del Negro et al., 2018), so their role could also be considered indirect. Future investigations will determine whether and to what extent any of the abovementioned structures participate in generating and/or sustaining the detected 0.2232 Hz rhythm. At this stage, it is worth noting that, despite appearing in the cortex, the new phase shift rhythm was detected during spontaneous breathing, implying that the breathing process was not influenced by an active and conscious subject. How and whether this rhythm will be modified during paced breathing remains an important question to be answered in our future experiments.

A major limitation in the present work is the fact that we did not record both respiratory and EEG signals from the same subjects simultaneously and consequently have not been able to prove that the detected *CFPS*(*t*) oscillation at ≈ 0.22 Hz was phase-locked to the respiration. Understandably, this is our first objective for future research. However, according to our view, the reasonable congruence of the recorded frequencies between the two subject groups participating in two different sets of experiments, as shown in Figures 9C, D, leaves little doubt that the detected *CFPS*(*t*) oscillation originates from the subjects’ respiratory activity. However, respecting scientific rigor, we abstained from naming it “respiratory rhythm” but rather used the term “respiratory frequency rhythm” in the title and throughout this work.

### 4.3 Alpha activity and respiration in different states of vigilance

If we compare the topographic distribution of five channel pairs with maximal FFT (*CFPS*(*t*)) oscillation amplitudes at 0.2232 Hz in wake and drowsy states (Figures 10B, 12B), one can see that in the wake state, they are predominantly longitudinal and laterally asymmetrical, positioned on the right side of the scalp. This particular distribution indicates that neural networks and populations positioned under and/or connecting anterior (frontal, F3, F4, and F8) and right posterior (P4, T6, and O2) scalp areas act in an organized way (underlying mechanisms unknown and remain yet to be elucidated) to participate in the respiratory frequency modulation of the detected alpha phase shifts. Considering the fact that neuronal excitability changes in synchrony with the instantaneous alpha phase (Romei et al., 2008; Sauseng et al., 2009; Mathewson et al., 2009; Berger et al., 2014), it follows that, for each channel pair, time delay of the excitation degree between these two large neuronal ensembles varies according to the respiratory frequency rhythm. This time delay, *Δt*, depends on the carrier frequency of the alpha activity, *f*_*∝*_ (≈10 Hz), and the instantaneous value of *CFPS*(*t*), Δφ: $\Delta t=\frac{\Delta \varphi \frac{1}{f_{\propto }}}{360{}^{\circ}}$ . Therefore, for each 10° of *CFPS*, *Δt* ≈ 2.78 m (compared to the *CFPS* waveform shown in the inlet of Figure 9C). The physiological significance of this rhythmically varying time delay is also not clear, but it obviously involves a more complex underlying mechanism related to, among others, the state of alertness and cognitive processes. However, since subjects did not perform any particular tasks, the topographic distribution of this rhythmicity in neuronal excitability may be more related to a state of readiness for incoming tasks, a kind of “phase shift resting state.” What appeared to be evident was that the large-scale respiratory frequency rhythmical integration of neuronal ensembles, involving anterior and posterior regions, seemed to exist in the wake state (Figures 10A, B). However, this picture was significantly altered in the drowsy state, where frontal regions appeared not to be rhythmically coupled with the posterior regions (Figures 12A, B). In terms of rhythmicity, anterior (frontal) and posterior regions became disentangled. The role of the frontal and prefrontal cortices across different states of alertness was already known (Muzur et al., 2002; Brown et al., 2012). Muzur et al. stated in their study that “functions of the prefrontal cortex are more relevant to the self-conscious awareness that is lost during sleep.” Similar results, pointing to the biggest amount of changes occurring in the frontal regions, were obtained in our previous analyses performed on the same set of signals during the wake to drowsy transition (Kalauzi et al., 2012b; 2015). Figures 10C, 12C present an additional result regarding the difference between the two states of vigilance: amplitudes of respiratory frequency *CFPS*(*t*) oscillations are greater in the wake state than those in the drowsy state, and this is valid for all channel pairs. Therefore, full wakefulness appeared to be necessary for this alpha phase shift oscillations to appear in their full capacity.

### 4.4 Volitional control of breathing and alpha phase shifts

It was found that, besides the cortex, breathing rates can be influenced by stimulation of the hippocampus, amygdala, and insula (Frysinger and Harper, 1990; Harper et al., 1998; Herrero et al., 2018), indicating a complex network of different brain structures during the volitional control of breathing. In contrast, scalp EEG (Fumoto et al., 2004), as well as TMS (Locher et al., 2006), and neuroimaging techniques (McKay et al., 2003) revealed the role of premotor, motor, and supplementary motor cortices in the process. All results presented in our work were obtained in the regime of spontaneous breathing. Within this experimental framework and at this moment, it is not possible to predict how these results, specifically profiles (oscillatory content) of *CFPS*(*t*) spectra and topographic distribution of their most prominent amplitude peaks, would appear within a paced breathing paradigm. Potential appearance of peaks at the frequencies of paced breathing would certainly strengthen the hypothesis that it is linked to the breathing process and point to its possible role in the volitional control. Nevertheless, in order to elucidate the effect of volitional control, future experiments using topographic analyses of *CFPS*(*t*) respiratory frequency amplitudes under paced breathing should take care to place scalp electrodes over the cortical structures mentioned above.

Regarding the results of statistical tests presented in Figure 13, we found that, although amplitudes of alpha activity are reduced in the drowsy state compared to the wake state (Bojić et al., 2010), *CFPS*(*t*) oscillations did not disappear but still persisted at the frequency of 0.2232 Hz (although also reduced; Figures 10C, 11D, 12C). However, let us point out that we obtained an unexpectedly high number of channel pairs (20/91), which exhibited respiratory frequency amplitudes significantly larger in wake than in the drowsy state. To have 22% of such pairs, with only 10 subjects analyzed, seems encouraging and raises expectations that future experiments, if performed on a wider group of subjects, might produce more information and offer new insights into this newly discovered oscillation. For example, since our results point to a ≈0.2-Hz rhythmical variability of time delays between occurrences of extremal (minimal or maximal) neuronal excitability between different scalp regions, with simultaneously recorded EEG and respiratory signals, we would be able to determine whether these delays are (or not) locked to extremal values of the neuronal excitability caused by respiration, allowing us a clearer and more detailed picture of the interaction between respiration and alpha waves. In addition, a high percentage of channels with significantly different FFT respiratory frequency amplitudes between the two states of vigilance points to an important role of wakefulness and conscious awareness in the dynamical organization of respiratory modulation of alpha brain activity.

### 4.5 Network approach: how to connect the brain, respiration, and cardiac activity

Cardiac rhythm and its causal relationship with EEG were studied in different sleep stages by different methodological approaches (e.g., by Granger causality; Abdalbari et al., 2022). In the course of this study, we searched for the presence of cardiac-like rhythm (around 1 Hz) in our FFT (*CFPS*(*t*)) spectra but could not detect it in any pair of electrodes. In contrast, in the case of simultaneous EEG and respiratory recordings, *CFPS*(*t*) time series may be included into multivariate predictability analyses such as those performed by Faes et al. (2016) in different sleep or pathological experimental paradigms (apnea-related conditions are particularly interesting states to explore).

Another set of situations where phase relations between respiration and *CFPS*(*t*) could be studied comprises different emotional psychological states since they involve both the brain and cardiovascular systems. It was shown that emotional response is initially caused by sympathovagal activity, in which ascending modulations precede efferent information transfer and correlate with the level of arousal (Candia-Rivera et al., 2022).

In course of the brain–body interaction studies, a network approach was employed involving multivariate correlation analysis (Pernice et al., 2021) and information dynamics (information storage and information transfer; Zanetti et al., 2019). More relevant to us was the work of Pernice et al., who calculated multivariate correlations within and between the human brain–body subnetworks in three experimental conditions representing different levels of mental stress: rest, sustained attention, and mental arithmetic. The quantities of interest to our present approach were the heart rate (η), respiration amplitude (ρ), and amplitudes of four EEG bands: δ, θ, α, and β. The authors found that internal subnetwork linkages were most common (mainly η–ρ; δ–θ; θ–α; and α–β). However, two inter-subnetwork correlations were also significant (η–β and η–δ). Therefore, when only amplitudes were being studied, neither alpha nor respiration was found to be mutually correlated. It would be an interesting alternative to probe whether alpha phases (phase shifts) are correlated with respiration phases in these states of *heightened* alertness as we found a considerable topographic redistribution of channels with maximal alpha FFT (*CFPS*(*t*)) peaks at 0.2232 Hz in the state of *reduced* alertness (drowsiness), compared to the nominal state (resting wakefulness).

Spontaneous breathing induces diverse autonomic rhythms that modulate cardiovascular control including arterial blood pressure and cerebral blood flow—heart rate and stroke volume— which, in turn, can influence EEG oscillations (Rau et al., 1993; Liu et al., 2019). Respiratory sinus arrhythmia, which has been intensively studied for decades (Hirsch and Bishop, 1981; Larsen et al., 2010), is an important part of this rhythmicity. All of the abovementioned rhythms initially originate from the breathing process itself, although the situation is more complex, as studies of causal relationships between cardiovascular and respiratory systems revealed a bidirectional influence (Tzeng et al., 2003; Cairo et al., 2023; Platiša et al., 2023; Porta et al., 2024). Therefore, having this network complexity in mind, if our hypothesis were to be proven, it would be a challenge to explore the role of RSA in the causal chain of these rhythmic processes. One possible approach might be to search for the link/correlation between the amplitude of RSA and FFT (*CFPS* (*t*)) peak height at ≈ 0.22 Hz in various experimental conditions and different physiological states.

Cardiorespiratory variables like the respiratory rate, heart rate, systolic arterial pressure, mean arterial pressure, and mean cerebral blood flow are main candidates for mediators by which the brain and respiration exert mutual influence. For instance, it has been shown that respiratory activity can act as a confounder or suppressor of the causal relationship between different cardiovascular and cerebrovascular variables. By applying the confounding/suppression test on propofol-based general anesthesia patients and controls, Porta et al. (2022) concluded that respiration behaved systematically as a confounder for cardiovascular and cerebrovascular controls. Causal relationships between the abovementioned variables can be quantitatively assessed by applying well-established methods both in time (Granger, 1980) and frequency (Geweke, 1982) domains. By studying postural syncope propensity, Porta et al. (2023) found that the postural syncope is favored by a loss of coordination between the baroreflex feedback and mechanical feedforward pathway in response to head-up tilt (HUT) in the LF band and by a weaker ability of cerebral autoregulation to limit mean cerebral blood velocity variability driven by mean arterial pressure changes in the respiratory rate during HUT. Having these results in mind, it cannot be *a priori* excluded that the respiratory impact on alpha phase shifts is mediated through pressure-to-flow at the cerebral level and baroreflex at the respiratory rate. Moreover, the relationship between the cerebral blood flow and alpha EEG activity (especially their phases; see Jann et al., 2009) must be modeled quantitatively in order to complete this cause–effect chain. Hopefully future analyses, specifically Geweke spectral causality applied to *CFPS*(*t)* and other abovementioned simultaneously recorded variables (primarily respiration), in spontaneous and paced breathing, will shed some new light on the causal relationships in the respiratory frequency range within and between respiratory, cardiovascular, and cerebrovascular systems. A carefully designed experimental setup, able to assess possible concomitant influences from respiration to EEG alpha activity (and *vice versa*), by mean arterial pressure to mean cerebral blood flow and to the heart period through baroreflex, at the frequencies of respiration and heart rate (respectively), is necessary.

### 4.6 Potential future use of the detected rhythm

It is known that changes in cardiorespiratory coupling (CRC) are most expressed in stressful conditions. In some pathological states, such as type 2 diabetes mellitus, it was shown that they could be used as an early marker of cardiac autonomic dysfunction (Da Silva et al., 2023). The other set of conditions where CRC becomes increasingly strained involve physical exercise and sports training, where universal methodological standards have not yet been fully established (de Abreu et al., 2023; de Abreu et al., 2024). If our hypothesis is to be confirmed, we would have a newly detected EEG rhythm, probably more firmly correlated with respiration than existing EEG rhythms, in addition to conventional EEG–ECG–respiration recordings. In that case, present CRC could be extended to cardiorespiratory–brain coupling (CRBC), making it more sensitive to the changing conditions or pathological situations such as tumors, multiple sclerosis, or even degenerative diseases. We expect that in future experiments with *CFPS*(*t*)–ECG–respiratory signals, additional information could be acquired to enable us to reach those standards more easily.

## Data Availability

The raw data supporting the conclusions of this article will be made available by the authors, without undue reservation.
